# Supplementary material for: Exploring the clinical context of adopting an instrumented insole: a qualitative study of clinicians’ preferences in England
Source: BMJ Open. 2019 Apr 20;9(4):e023656. doi: 10.1136/bmjopen-2018-023656 (PMC6500285; doi:10.1136/bmjopen-2018-023656)
Supplement: Supplementary file 1 [file bmjopen-2018-023656supp001.pdf]

**Semi-structured interview questions for clinicians**

**Contextual Background**

- What is your age and nationality (you can prefer not to say)?
- What is/can you describe your professional role?
- How long have you been in this role?
- What types of patients do you come into contact with?
    A. Is there a particular type/category e.g OA/ACL?
    B. Does it vary?
    C. How many patients do you see per day?
    D. Is there a particular age range of patients you work with?

**Wearable Technologies**

- What do you know about wearable technologies?
- Do you currently use any wearable technologies in your work?

**Clinician Preferences for the Flexifoot Device (With Reference to OA Surgery and ACL Injuries)**

- Would you find Flexifoot useful for your OA and/or ACL injury patients?
- How would such a device help you in your own work? How would you use it?
- What would you specifically like to measure using the Flexifoot device?
- Would you like information about e.g.
    A. the patient's gait, such as symmetry/stride length?
    B. centre of pressure and pressure profiles for subsections of the foot?
    C. ground reaction force?
- Is there any parameters that should be tailored for OA surgery or for ACL injuries patients? (Different measurements used in accordance to the patients?)
- In addition to specific parameters, would you find it useful to measure the activity level of your patients? (e.g. time when active or not)
- How do you feel about monitoring compliance to exercise programmes?
- How often and for how long would you want the patient to wear the device?
- How would you like the data presented to you? E.g. in graphs, summary tables, performance profiles over a certain period of time?
- Would you find it useful to have a brief summary of the patient's progress with the option to look in more detail at certain aspects of the data?
- When would you like the data to be available to you? E.g. every day the patient uses Flexifoot?
- How would you ideally access the data? Would you like it emailed to you or would you prefer to have a website where you can log in to access it?
- Would you find an automatic alert system useful that told you when the data was available from your patient?
- Would you like an alert that will flag up if the metrics you identified fall below a certain threshold? (e.g. the patient is not doing exercise at all, so you need to send them a reminder?)
- Would you find Flexifoot to be more useful for patients with ACL injury or for pre-/post-OA surgery?
- What other information would you use to complement the use of Flexifoot in your clinical practice? Any other parameters that you think will be valuable that cannot be measured with Flexifoot?
- Can you suggest any reasons that may prevent you from using this device should it became available for use in clinical practice?

**Closure**

- Do you have any other comments about the Flexifoot device?
