# Supplementary material for: Exploring the clinical context of adopting an instrumented insole: a qualitative study of clinicians’ preferences in England
Source: BMJ Open. 2019 Apr 20;9(4):e023656. doi: 10.1136/bmjopen-2018-023656 (PMC6500285; doi:10.1136/bmjopen-2018-023656)
Supplement: Author's manuscript [file bmjopen-2018-023656.draft_revisions.pdf]

BMJ Open

BMJ Open is committed to open peer review. As part of this commitment we make the peer review history of every article we publish publicly available.

When an article is published we post the peer reviewers' comments and the authors' responses online. We also post the versions of the paper that were used during peer review. These are the versions that the peer review comments apply to.

The versions of the paper that follow are the versions that were submitted during the peer review process. They are not the versions of record or the final published versions. They should not be cited or distributed as the published version of this manuscript.

BMJ Open is an open access journal and the full, final, typeset and author-corrected version of record of the manuscript is available on our site with no access controls, subscription charges or pay-per-view fees (http://bmjopen.bmj.com).

If you have any questions on BMJ Open's open peer review process please email info.bmjopen@bmj.com

BMJ Open

Exploring the clinical context of an instrumented insole: Is there scope for clinical adoption?

Journal:	*BMJ Open*
Manuscript ID	bmjopen-2018-023656
Article Type:	Research
Date Submitted by the Author:	17-Apr-2018

Complete List of Authors:	Lin, Denise; Imperial College London Department of Surgery and Cancer<br>Papi, Enrica; Imperial College London, Department of Surgery and Cancer<br>McGregor, Alison; Imperial College London, Dept of Surgery and Cancer

Keywords:	wearable technologies, Osteoarthritis, Self-managment, Pressure sensors, Users' preferences

SCHOLARONE™
Manuscripts

For peer review only

Exploring the clinical context of an instrumented insole: Is there scope for clinical adoption?

Denise Lin[a], Enrica Papi[a,b], Alison McGregor[a]

[a]Department of Surgery and Cancer, Imperial College London, London, UK
[b] Department of Bioengineering, Imperial College London, London, UK

Corresponding author:
Dr Enrica Papi
Department of Surgery and Cancer,
Imperial College London,
Room 7L16, Floor 7, Laboratory Block,
Charing Cross Hospital,
London, W6 8RF, UK
Email: e.papi@imperial.ac.uk
Phone: 020 3313 8833

Denise Lin: email: denise.lin14@imperial.ac.uk

Alison H McGregor: email: a.mcgregor@imperial.ac.uk

ABSTRACT

Objectives: The study explores clinicians' views of the clinical uptake of a smart, flexible, pressure-sensing insole, named Flexifoot, to enhance the care and management for patients with osteoarthritis (OA). Clinicians are key users of wearable technologies, and can provide appropriate feedback for a specific device, such as Flexifoot, for successful clinical implementation.

Design: Qualitative study with in-depth, semi-structured interviews, analysed using deductive analysis generating key themes.

Setting: The study was conducted in a University setting.

Participants: 30 clinicians were interviewed, including 11 physiotherapists, 11 orthopaedic surgeons, 5 general practitioners and 3 podiatrists.

Results: All clinicians regarded the wearable technology, Flexifoot, to be useful for the long-term monitoring of patients objectively, in adjunction to current methods. The data obtained may be beneficial for enhancing information exchange between clinicians and patients, and also between clinicians themselves. The data may provide useful feedback to clinicians and patients, and enables its use for rehabilitation and screening. Feedback and self-monitoring by patients may motivate them as they can set goals for themselves, and may reduce costs of unnecessary clinic visits. The data interface should be secure, concise and visually appealing for all. The measured specific parameters of Flexifoot, its duration of wear and the frequency of data output would all depend on the rationale for its use. The clinicians and patients must collaborate to optimise the use of Flexifoot for the long-term monitoring of disease for patient care in clinical practice.

Conclusions: The wearable insole may complement and improve current methods of long-term patient management for OA or other conditions in clinical settings. The role of Flexifoot may be useful for objective measures and should be tailored carefully on a case-by-case basis. Adopting the device, and other similar technologies, requires reducing the main barriers to use (time, cost, patient compliance) before its successful implementation.

Keywords: wearable technologies, osteoarthritis, self-management, users preferences, pressure sensors

Article Summary

- This was the first to specifically refer to implementing the use of an in-house smart, flexible, pressure-sensing insole tool into clinical practice for patients with osteoarthritis
- The views of clinicians were fully explored with in-depth interviews, giving rise to detailed suggestions to optimise the device's role alongside current strategies in patient care
- Clinicians were unable to use the device themselves prior to the interviews and responses were based upon one single demonstration and explanation of the tool
- Findings of this study were based on clinicians' perspectives who initially had a varied level of experience and familiarity of wearable technologies between them

INTRODUCTION

Osteoarthritis (OA) is one of the most common long-term musculoskeletal diseases, cause of pain and functional disability[1]. Individuals who have sustained a knee injury, such as anterior cruciate ligament (ACL) injuries, are 3-6 times more likely to develop knee OA[2–4]. In these patients, diagnosis occurs approximately 10 years prior to those without a previous injury[3,4], calling for long-term management of such conditions. Current clinical guidelines recommend physical activity to delay surgical intervention that has limited lifespan and poor reported patient outcomes[5–8]. Conversely, poor patient compliance limits long-term exercise benefits for OA.

Pain and functional gait changes are reasons why OA patients primarily visit clinicians. Gait analysis helps to establish OA diagnoses, severity and biomechanics underpinning musculoskeletal disorders[9]. In clinical settings, however, patient gait is observed by the clinician eye, and self-reported questionnaires, such as the *36-Health Survey (SF-36)*, *Western Ontario and McMaster Universities Osteoarthritis Index (WOMAC)*, *Oxford Knee Score (OKN)* and *Knee Osteoarthritis Outcome Scores (KOOS)*, can assess OA severity[9–11]. Gait monitoring through clinician observation and patient questionnaires are prone to subjective responses, and therefore, are inadequate methods to quantify symptoms.

The emergence of wearable technologies can enhance current tools of physiotherapy, rehabilitation and daily monitoring of physical activity. Novel, portable wearable technologies offer a promising approach for use outside of the laboratory, to monitor functional changes in disease progression and activity levels. Nevertheless, the clinical implementation of wearable technologies is seemingly difficult. To enhance translation into clinical context, patient and clinicians' preferences have been explored in the past to determine the views and criteria of users for wearable technologies[12]. OA patients revealed that tracking disease progress was appealing and encouraged exercise[13]. Thirteen clinicians supported wearable technologies for their patients with OA clinically, but the preferences provided did not have specificity to one device[14].

Within our group, we developed a smart, flexible, pressure-sensing insole, aptly named 'Flexifoot'. Flexifoot generates plantar pressure readings from various foot regions. A high-resolution pressure map can be created from data that feeds back wirelessly to a smartphone app, for the extrapolation of gait spatio-temporal parameters, centre of pressure and pressure distribution. Flexifoot, being portable, in contrast to laboratory based-force-platforms, allows for continuous data collection over a substantial number of gait cycles, for feedback to patients and clinicians as needed. Daily gait and pressure analysis can enable patients to monitor improvements and disease-related progression, as well as guide clinicians through treatment decisions.

The ambiguity of previously obtained clinician preferences lacks the definitive feedback required to improve the design of a specific tool. The lack of specificity can be addressed by probing more into the details of the clinical implementation of Flexifoot for OA and other disorders. The diagnosis and management of OA involves a multitude of healthcare professional types and it is therefore important to understand how Flexifoot could best address their requirements to stir design and outcome measures towards clinical uptake. The aims of this study were to explore the clinicians' preferences for their use of Flexifoot and to identify specific parameters to be measured by the tool to enhance OA patient care.

For peer review only - http://bmjopen.bmj.com/site/about/guidelines.xhtml

METHODS

Study Design

The study was a qualitative study based on in-depth semi-structured interviews with 30 clinicians.

Participants

30 clinicians (18 males and 12 females, aged 21-57), including 11 physiotherapists, 11 orthopaedic surgeons, 5 general practitioners (GPs) and 3 podiatrists, were recruited for one-to-one interviews. The recruitment, via telephone and email, began from October 2015 and interviews occurred until September 2017.

The healthcare professionals practiced amongst private and National Health Service (NHS) settings within London and Greater London, one in Hereford, one in Cheltenham and one in Liverpool. They had from 4 months up to 28 years of experience within current specialities.

Interviews and Data

The interviews were performed by researchers (DL and MG) in person, except for 2, which were conducted over the telephone due to scheduling constraints. Face-to-face interviews were audio-recorded and transcribed afterwards.

Prior to each interview, participants' consent was obtained and researchers explained project aims, described Flexifoot and showed a prototype to each clinician (except in telephone interviews). Open-ended questions prompted clinicians to explore their perspectives regarding the relevance of Flexifoot in their clinical practice. The interview questions (Figure 1) highlighted Flexifoot's clinical influence, specific measurements, data presentation preferences, and gave scope for feedback and improvement.

The interview verbatim transcriptions were analysed using deductive thematic analysis[15], and key findings were collated into appropriate themes by DL, MG and EP.

RESULTS

The semi-structured interviews opened with questions to determine healthcare backgrounds of clinicians and the relevance of wearable technologies within their profession. 24 out of 30 clinicians were aware of wearable technologies, and 4 used them for patients.

Deductive analysis[15] of interview responses revealed four main themes, with sub-themes: use (applications, specific measurements, duration of wear), data presentation (data access, visual presentation, frequency of data), barriers to use and future development. The former three themes surfaced from specific interview questions asked. The latter was brought about after clinicians offered feedback as to how Flexifoot could be improved. The themes will be hereinafter described and verbatim quotes are indicated by: PT (physiotherapists), OS (orthopaedic surgeons), P (podiatrists) and GPs (general practitioners), followed by randomly assigned numbers.

Uses

Applications

The main uses of Flexifoot identified by clinicians are shown in Table 1.

Table 1: The main five applications of Flexifoot in clinical practice identified by 30 clinicians

**Main Applications of Flexifoot in Clinical Practice**
• Assessing efficacy of treatment (pre- and post-treatment)
• Monitoring disease progression
• Feedback for patients and other clinicians

•	Monitoring activity levels and compliance
•	Screening test to support future management

All groups of clinicians recognised Flexifoot as an objective outcome measure tool to monitor various parameters. 23 clinicians expressed that Flexifoot objective measures are useful to assess symptoms, and progress before and after medical intervention.

"It would be useful as an objective outcome measure for change…assess patients at time intervals for pre- and post-surgery." PT7

"This would be very interesting for research or pre- and post-surgery because you'd be able to monitor and look at change…it would definitely be useful as a follow-up guidance to surgical correction." OS11

21 clinicians felt that objective data can reinforce clinical interpretations and enhance information exchange between healthcare professionals. Moreover, real-time objective feedback can help to visually demonstrate the problem and solutions to patients.

"For us feeding back to the surgeons…you can be a bit more accurate about what it is that you're saying." PT11

"It might be useful to demonstrate to the patient what some of their symptoms are. To give them a visual representation of that, I could show them this while they're walking." OS10

"It's important for the patients to visualise what the problem is…as a relatively low-grade, without major intervention, you could do a lot with it to see how to correct problems objectively…It may then allow them to see visually what the issue is, so that if they correct it with the help of someone." GP2

Clinicians recognised Flexifoot as a self-management tool: rehabilitation targets can be set by patients themselves, or by clinicians, and patients become motivated.

"For patients to use at home as a rehabilitation tool to set targets or goals they can monitor themselves." PT8

"Anything that can give feedback to the patient themselves, to become more active and more healthy, then that could be of benefit, not necessarily to me, but to the patient." OS9

75% of the clinicians strongly supported Flexifoot measuring compliance to clinical advice.

"It would be really useful in terms of actually keeping a diary of what your patients are doing, especially with the osteoarthritis patients." PT5

"It is helpful if you have any doubts as to whether the patients are being compliant, if they are doing too much activity or too little activity." OS10

Clinicians can use the feedback as a screening tool and to help determine next steps for patient management.

"You could use that as some kind of screening test…do they really need to have a knee replacement yet?" OS1

"In conjunction with the physiotherapists, so if you were trying to get them to do a particular rehabilitation programme. Monitor what they're doing, that might be very useful." OS6.

However, GPs felt that Flexifoot was a tool to be used more by patients, rather than by GPs for planning patient care: *"an intervention that's positive for the patient, as opposed to this being an investigation" GP2,* and that feedback would be better interpreted by clinicians with greater musculoskeletal knowledge.

"I can't see any acute use for it that's going to change the patient's management right this second now." GP2

"I'm not sure whether this would change my management for the conditions." GP5

For peer review only

Moreover, various participants in all clinician groups agreed that Flexifoot may be more effective not as a stand-alone device, but to enhance current methods for diagnosis and prognosis, as an *"adjunctive thing to what I already have" P3*.

Specific Measurements

Due to the vast array of parameters that can be analysed after Flexifoot use, it was important to determine the most clinically appropriate specific measurements. Participants were prompted by suggestions of symmetry, stride length, centre of pressure, pressure profiles or ground reaction force. The ideal specific measurements differed slightly between clinician groups due to varying levels of knowledge, but there was a convergence of agreed parameters to be measured by Flexifoot in all clinician groups (Figure 2). GPs provided fewer preferable specific measurements due to gaps in their knowledge regarding musculoskeletal disorders.

The clinicians who supported the monitoring of activity levels (type and number of steps) demonstrated its importance for non-OA conditions too.

"It would give you an idea of their daily routine and their exercise levels and things, particularly if they've got other conditions such as diabetes or cardiovascular disease which can be improved by exercise." GP3

Two clinicians exhibited a cautious view regarding specific measurements when tracking patients' activity outside of clinical environments:

"You've got to be careful with monitoring… you don't want engender this sort of 'we're watching you' big brother idea." PT3

"I do think that there could be some, a little degree of patients being suspicious of you checking up on them and they may question the clinician – why can't you trust what you're telling them, why do they need to see what I'm doing?" GP2.

Duration of Wear

The duration of Flexifoot wear suggested by clinicians varied from single usage at clinic appointments, up to long-term periods of over one year, implying a range of uses. The nature of the tool being able to offer real-time advice allows for its acute use in the clinic. The prolonged use of Flexifoot may be appropriate for patients with chronic diseases or post-operatively. The disparity between preferred lengths depended on the rationale for patient use. 7 clinicians revealed that the duration of Flexifoot wear would be dependent on what outcomes were to be achieved (PT1, PT6, PT8, P2, OS1, OS6), type of injury (PT10), and patient age and compliance (GP5).

"If we were using it as a 'how do they move', we want a picture of their footprint, 5 minutes as they do a walk along the corridor." PT1

"During periods of activity, if they're experiencing pain or there is a particular challenging part of their day…short bursts of time which are critical to look at, so definitely not all day." OS3

However, long-term monitoring was preferred by most clinicians, where data collection would span over days, weeks, months and years, or *"for as long as it took to establish a meaningful change" PT3.*

"For 24hours or a few days…you'd want the results of this to reflect what they normally do." GP3

A short period of time using the device but at longer intervals between uses was also suggested:

"Snapshots at certain periods, much like we do at follow-up, at 6 weeks, 3 months, and a year." OS4

To maximise the personalised use of Flexifoot, clinicians and patients must collaborate to choose an appropriate duration of wear for each particular case.

Data Presentation

Data Access

For peer review only

50% of the clinicians would prefer to access the data by logging into a system, and some expressed that it should be integrated amongst patients' current notes for information crossover.

"If there was a way of tying in the results so that when you log in and click on the patient, it came up on their results. That would be the most ideal way." GP3

5 clinicians preferred the data via email, although 3 clinicians stated that maintaining patient confidentiality was paramount.

"Emailed is easier, but then log-in would be better because safety aspects and confidentiality." P2

Visual Presentation

The data should be concise so that clinicians can quickly interpret data. The clinicians expressed differing opinions after they were prompted with suggestions of graphs, tables and pictures. Therefore, numerous display options should be available to accommodate for all preferences.

"We need to have a summary that is brief, because you don't want to look through tons and tons of data. Then, if you wanted to look into more detail, then there should be the option." GP4

The data should be easily comprehended by patients too. A visually appealing approach with an additional colour-system scheme can enhance patient understanding.

"Patients want to know as well, they want a variation that's patient friendly…an easier format for patients to understand." OS6.

The addition of normal reference ranges alongside the objective measures allows for the comparison of patient parameters versus reference data; this will enhance user-experiences and allow for goals to be set.

"Patient specific – it would be graphs. It would be nice to have a normal distribution and see where they fit inside the normal distribution." OS9

Frequency

The clinicians want to receive and access data at appropriate times, such as immediately before or during appointments. The majority (28/30) of clinicians were unlikely to monitor patients outside of the clinic due to time-restraints.

"I'd access the data just prior to the patient coming in or during that appointment. We would only really have time to monitor or review the data when the patient is actually here." PT5

"Real-time is helpful for the patient in a therapy session. If they had information once a week on how they're doing. Alerts are ideal, but there is no time to check it." PT10

The generation of data should occur in a timely fashion, *"correlated with the patient's clinic appointment" OS9.* The frequency of data received should depend on personal clinician preferences and the purpose of Flexifoot use.

"There should be an option for the data to be accessible when you want it and you choose." GP5

Barriers to Use

Clinicians highlighted obstacles to implementing Flexifoot clinically (Table 2).

Table 2: The barriers to the use of implementing Flexifoot in clinical practice identified by 30 clinicians

**Barriers to the Use of Flexifoot**
• Time
• Cost and availability
• Influence on practice
• Training/education required

For peer review only

• Patient compliance
• Hygiene control

50% of the clinicians illustrated that time restraints were the largest concern of Flexifoot use in clinical practice, including time taken for initial patient assessments, device introduction, and data generation.

"For me to use this for one patient, explain how to use it and monitor their activity is probably unlikely and unrealistic given the general practice workload and increasing demands on GPs." GP2

"That is the difficulty with this, it is an additional investigation that we need to spend time assessing." OS11

The second most identified barrier was cost – *"it has to be suitably priced…that an average practice can afford" P1.*

The inability of clinicians and patients to interpret data and the training required is another issue. 5 GPs stressed that Flexifoot was too specialised for their environment.

"Patients might not understand the data…people are not familiar with technologies, but this will be less of a problem in the future." PT9

"If it's mainly biometrics and gait analysis…I don't think that I would see this as being within a GP's remit so much. The biggest barrier for me not using it is, identifying how the information it gives would fall into my remit, and how it would influence my practice". GP3

Poor patient compliance also hinders Flexifoot's prospective use.

"Patients are so unreliable and I wouldn't be confident that they would remember to transfer it to another pair of shoes or if they take their shoes off and we'd not be tracking anything." OS3

6 clinicians identified hygiene and infection risks if the device was used for long periods, or between different patients. Flexifoot use between multiple patients could be more economically practical however.

"I suppose you've got questions of hygiene…you'd need a material you can wipe and maybe some way of cleaning them really well, so they would be to an infection control standard. If you can use them more than once, it would be cost effective." P3

Future Development

Following the responses to the set questions, clinicians offered suggestions on how to optimise Flexifoot for a successful clinical uptake.

Parameters that would be ideally measured using Flexifoot (Figure 2) could be adapted to measure more factors and expand patient target audiences. Clinicians suggested the use of Flexifoot in Parkinson's disease, peripheral neuropathy in diabetes, chronic pain conditions, obesity, hemiplegia and tendinopathies.

"Pressure profiles are good, but it'd be really great to measure shear and temperatures in the foot…it would be better and more useful for people with plantar foot pain. Diabetics – it would be great." OS11

Design changes were also proposed, *"maybe this ribbon (ankle strap) could be a bit smaller because it might irritate someone on the side and it might artificially affect their gait" OS10.*

DISCUSSION

A technology must be user-friendly to optimise its efficacy and sustainability[16,17]. We conducted structured interviews with clinicians to guide the development of our novel technology, Flexifoot,

For peer review only

towards clinical uptake. The clinicians expressed numerous advantages of adopting Flexifoot into healthcare settings and barriers, indicating strategies for future improvement. The main advantage recognised by clinicians was the ability of Flexifoot to generate quantitative data, that can be used for monitoring and feedback in various clinical contexts.

Clinicians implied that Flexifoot would not replace current clinical tools, but instead complement them. The ambulatory quantitative data can support existing OA diagnostic and management tools, and help to improve the reliability of clinical decisions. The ambulatory monitoring of disease progression, alongside patients' responses to treatment, has been considered useful[18]. Other rehabilitation technologies that motivate and offer objective feedback have been associated with long-term benefits and good physical fitness levels[19]. Moreover, objective feedback can enable a more efficient exchange and handover of patient information between clinicians[20]**.** A clinician may use the tool as a screening approach in adjunction to current methods, for more reliable results and subsequently refer the patient onto a specialist[14]. Also, the clinicians recognised that the data can reinforce their dialogue with patients, making patients more aware of their problem. Awareness and feedback was seen as a way to enhance patient self-management. Tracking activity levels and feedback engages patient involvement in their own care, and is useful for other non-musculoskeletal chronic conditions too[21,22]. In relation to this, clinicians indicated how Flexifoot could be a tool for patients. The data being available to patients allows for greater independence in self-monitoring and feedback of their own diseases in familiar environments outside of clinics[18]. Home-based training and monitoring devices showed higher patient satisfaction compared to similar care within clinics[23]. Self-management can also reduce economic burdens as the technology can educate OA patients, improve outcomes and hence reduce hospital visits[18,24]. The extensity of uses and specific measurements suggested by clinicians was greatly dependent on the type of clinician and specific patient cohorts, which is also the case for other musculoskeletal interventions[25]. The GPs felt that they had insufficient gait analysis knowledge, and that the device was presently too specialist for general practice. GP environments may be inappropriate since it comprises of too broad a range of patients. Instead, they indicated that Flexifoot would be better suited for clinicians that follow up patients more regularly. This was reiterated by physiotherapists, orthopaedic surgeons and podiatrists, who are better equipped in musculoskeletal fields, and expressed positivity for the clinical adoption of Flexifoot.

All clinicians indicated that the system and data output should be easy to use and interpret. Ease of use of wearable sensors was reported in another study to facilitate their adoption into clinical settings for clinicians[26]. The prospect of a log-in software system for Flexifoot data was more popular than receiving results via email. The interface should be integrated alongside current patient records for information crossover and a choice of data presentation styles should be available. The material should be presented alongside normal reference ranges, for comparisons and targets to be made. Data accessibility and presentation should be understood by patients too – this is key for patient acceptance and accessibility[27]. Clinicians' views obtained in another study recognised that shorter, simpler and more concise data as educational material is preferable for patients, but that detailed data should be fully available too[28]. This agrees with our findings: participants expressed the possibility of having access to more detailed data if needed, whilst avoiding scanning excessive data beyond their understanding. Full data measurements could be stored however for more skilled users in research settings[29].

Furthermore, shortcomings of Flexifoot were recognised by the clinicians. The clinicians' continuous workloads means that using real-time data from Flexifoot is only feasible prior to or during appointments in the presence of patients. The real-time data and automatic alerts may be more useful for patient users for receiving feedback, which continues to motivate them[13]**.** Clinicians are reluctant for the introduction of new tools because they can disrupt time-pressured practice schedules, and the time required to train them to use Flexifoot must also be considered[30]**.** The clinical efficacy of Flexifoot must be therefore fully established before clinicians can adopt it as a method worthy of appointment time. High costs also limit new technology implementation within health services. The current expense to manufacture one Flexifoot device is low, but one tool per patient may be economically impractical. The recycling and reuse of devices between patients may reduce costs, but increases hygiene and infection

For peer review only

risks, expressed by 6 clinicians. Introducing hygienic procedures prior to and after Flexifoot use could enhance its reusability and cost-effectiveness. Thus, the cost-effectiveness, with reference to current treatment guidelines, should be further investigated. Patient compliance is also an issue, but effective clinician-patient communication can determine patient cohorts supportive of self-management, and those more likely to adhere to using Flexifoot[13,14,31]. Clinicians can promote the relevance of sensor technologies for patients' care, and hence boost compliance[12,32]. The identified issues surrounding Flexifoot are apparent in other wearable technologies too[25]. The replication of problems between devices implies a necessity for new approaches in encouraging patients' compliance and appeal for novel strategies. The findings that emerged from this study can be translated to other similar technologies to promote their clinical uptake and foster new developments.

The study limitations involved the clinicians' varied levels of experience and familiarity of instrumented insoles, and that 27/30 interviewees were based in the London area. The clinicians had not used Flexifoot, but its function was demonstrated or described prior to interviews. Future studies would involve clinicians' use of Flexifoot beforehand. Moreover, clinicians who were well-informed around the subject were perhaps biased to initially participate in the interviews and express positivity. However, the interviews were confidential and honest feedback was encouraged.

In conclusion, the clinicians considered Flexifoot to be a useful tool that could be used in adjunction to current approaches, in a long-term, follow-up setting to support and improve patient care. The clinicians' preferences exhibited numerous ways in which Flexifoot can be useful for patients with OA or other conditions. The measured parameters should be selected according to patient-specific cases, and delivered in a concise manner through a secure interface. A choice of data outputs should be offered to cater for all users. The challenges of time, cost, infection control should be addressed, alongside the clinical efficacy and cost-effectiveness for the clinical adoption of Flexifoot and similar technologies.

Author Contributions: DL, EP, and AHM conceived and designed the study. DL carried out the interviews; DL and EP analysed and interpreted the data. DL and EP drafted the manuscript. All authors read, edited, and approved the final version of the manuscript. All authors had full access to all of the data in the study and can take responsibility for the integrity of the data and the accuracy of the data analysis. We would also like to thank MSc student Mary Goodwin for her contribution with data collection.

Funding: This research received no specific grant from any funding agency in the public, commercial or not-for-profit sectors

Competing interests: The authors declare that there are no conflicts of interest.

All authors have completed the ICMJE uniform disclosure form at www.icmje.org/coi_disclosure.pdf (available on request from the corresponding author) and declare: no support from any organisation for the submitted work; no financial relationships with any organisations that might have an interest in the submitted work in the previous three years; no other relationships or activities that could appear to have influenced the submitted work.

Ethical approval: The study was reviewed and approved by Imperial College London Ethics Research Committee. All participants gave written informed consent.

Data sharing: No additional data available.

References

1 Bennell KL, Hunter DJ, Hinman RS. Management of osteoarthritis of the knee. *Bmj* 2012;**345**:e4934–e4934. doi:10.1136/bmj.e4934

2 Simon D, Mascarenhas R, Saltzman BM, *et al.* The Relationship between Anterior Cruciate Ligament Injury and Osteoarthritis of the Knee. *Adv Orthop* 2015;**2015**. doi:10.1155/2015/928301

3 Muthuri SG, McWilliams DF, Doherty M, *et al.* History of knee injuries and knee osteoarthritis: A meta-analysis of observational studies. *Osteoarthr Cartil* 2011;**19**:1286–93. doi:10.1016/j.joca.2011.07.015

4 Driban JB, Eaton CB, Lo GH, *et al.* Association of Knee Injuries With Accelerated Knee Osteoarthritis Progression: Data From the Osteoarthritis Initiative. *Arthritis Care Res* 2014;**66**:1673–9. doi:10.1002/acr.22359

5 Jones CA, Voaklander DC, Johnston DW, *et al.* Health related quality of life outcomes after total hip and knee arthroplasties in a community based population. *J Rheumatol* 2000;**27**:1745–52.http://www.ncbi.nlm.nih.gov/pubmed/10914862 (accessed 29 Mar 2018).

6 Wylde V, Hewlett S, Learmonth ID, *et al.* Persistent pain after joint replacement: Prevalence, sensory qualities, and postoperative determinants. *Pain* 2011;**152**:566–72. doi:10.1016/j.pain.2010.11.023

7 Singh JA, O'Byrne M, Harmsen S, *et al.* Predictors of moderate-severe functional limitation after primary Total Knee Arthroplasty (TKA): 4701 TKAs at 2-years and 2935 TKAs at 5-years. *Osteoarthr Cartil* 2010;**18**:515–21. doi:10.1016/j.joca.2009.12.001

8 Dowsey MM, Nikpour M, Dieppe P, *et al.* Associations between pre-operative radiographic changes and outcomes after total knee joint replacement for osteoarthritis. *Osteoarthr Cartil* 2012;**20**:1095–102. doi:10.1016/j.joca.2012.05.015

9 Elbaz A, Mor A, Segal G, *et al.* Novel classification of knee osteoarthritis severity based on spatiotemporal gait analysis. *Osteoarthr Cartil* 2014;**22**:457–63. doi:10.1016/j.joca.2013.12.015

10 Harris KK, Dawson J, Jones LD, *et al.* Extending the use of PROMs in the NHS—using the Oxford Knee Score in patients undergoing non-operative management for knee osteoarthritis: a validation study. *BMJ Open* 2013;**3**:e003365. doi:10.1136/bmjopen-2013-003365

11 Roos EM, Roos HP, Lohmander LS, *et al.* Knee Injury and Osteoarthritis Outcome Score (KOOS)—Development of a Self-Administered Outcome Measure. *J Orthop Sport Phys Ther* 1998;**28**:88–96. doi:10.2519/jospt.1998.28.2.88

12 Bergmann JHM, McGregor AH. Body-worn sensor design: What do patients and clinicians want? *Ann Biomed Eng* 2011;**39**:2299–312. doi:10.1007/s10439-011-0339-9

13 Papi E, Belsi A, McGregor AH. A knee monitoring device and the preferences of patients living with osteoarthritis: a qualitative study. *BMJ Open* 2015;**5**:e007980. doi:10.1136/bmjopen-2015-007980

14 Papi E, Murtagh GM, McGregor AH. Wearable technologies in osteoarthritis: a qualitative study of clinicians' preferences. *BMJ Open* 2016;**6**:e009544. doi:10.1136/bmjopen-2015-009544

15 Ritchie J, Lewis J. QUALITATIVE RESEARCH PRACTICE A Guide for Social Science Students and Researchers. https://mthoyibi.files.wordpress.com/2011/10/qualitative-research-practice_a-guide-for-social-science-students-and-researchers_jane-ritchie-and-jane-lewis-eds_20031.pdf (accessed 13 Dec 2017).

16 Lewis JE, Neider MB. Designing Wearable Technology for an Aging Population. *Ergon Des* 2017;**25**:4–10. doi:10.1177/1064804616645488

17 Lee J, Kim D, Ryoo H-Y, *et al.* Sustainable Wearables: Wearable Technology for Enhancing the Quality of Human Life. *Sustainability* 2016;**8**:466. doi:10.3390/su8050466

18 Majumder S, Mondal T, Deen M. Wearable Sensors for Remote Health Monitoring. *Sensors* 2017;**17**:130. doi:10.3390/s17010130

19 Aamot I-L, Karlsen T, Dalen H, *et al.* Long-term Exercise Adherence After High-intensity Interval Training in Cardiac Rehabilitation: A Randomized Study. *Physiother Res Int* 2016;**21**:54–64. doi:10.1002/pri.1619

20 Popovici I, Morita PP, Doran D, *et al.* Technological aspects of hospital communication challenges: An observational study. *Int J Qual Heal Care* 2015;**27**:183–8. doi:10.1093/intqhc/mzv016

21 Chiauzzi E, Rodarte C, DasMahapatra P. Patient-centered activity monitoring in the self-management of chronic health conditions. *BMC Med* 2015;**13**:1–6. doi:10.1186/s12916-015-0319-2

22 Thornton JS, Frémont P, Khan K, *et al.* Physical activity prescription: a critical opportunity to address a modifiable risk factor for the prevention and management of chronic disease: a position statement by the Canadian Academy of Sport and Exercise Medicine. *Br J Sports Med* 2016;**50**:1109–14. doi:10.1136/bjsports-2016-096291

23 Kraal JJ, Elske Van Den Akker-Van Marle M, Abu-Hanna A, *et al.* Clinical and cost-effectiveness of home-based cardiac rehabilitation compared to conventional, centre-based cardiac rehabilitation: Results of the FIT@Home study. *Eur J Prev Cardiol* 2017;**24**:1260–73. doi:10.1177/2047487317710803

24 Bodenheimer T, Lorig K. Patient Self-management of Chronic Disease in Primary Care. 2014;**288**:2469–75. doi:10.1001/jama.288.19.2469

25 de Almeida PHTQ, MacDermid JC, Pontes TB, *et al.* Orthotic use for CMC osteoarthritis: Variations among different health professionals in Brazil. *J Hand Ther* 2016;**29**:440–50.

For peer review only

doi:10.1016/j.jht.2016.07.007

26 Grimm B, Bolink S. Evaluating physical function and activity in the elderly patient using wearable motion sensors. *EFORT Open Rev* 2016;**1**:112–20. doi:10.1302/2058-5241.1.160022

27 Puri A, Kim B, Nguyen O, *et al.* User Acceptance of Wrist-Worn Activity Trackers Among Community-Dwelling Older Adults: Mixed Method Study. *JMIR mHealth uHealth* 2017;**5**:e173. doi:10.2196/mhealth.8211

28 Grudniewicz A, Bhattacharyya O, McKibbon KA, *et al.* User-centered design and printed educational materials: A focus group study of primary care physician preferences. *J Contin Educ Health Prof* 2016;**36**:249–55. doi:10.1097/CEH.0000000000000112

29 Iqbal MH, Aydin A, Brunckhorst O, *et al.* A review of wearable technology in medicine. *J R Soc Med* 2016;**109**:372–80. doi:10.1177/0141076816663560

30 Yarbrough a. K, Smith TB. A new take on TAM. *Med Care Res Rev* 2007;**64**:650–72. doi:10.1177/1077558707305942

31 Loos JR, Davidson EJ. Wearable health monitors and physician-patient communication: The physician's perspective. *Proc Annu Hawaii Int Conf Syst Sci* 2016;**2016–March**:3389–98. doi:10.1109/HICSS.2016.422

32 Teh RC-A, Mahajan N, Visvanathan R, *et al.* Clinical effectiveness of and attitudes and beliefs of health professionals towards the use of health technology in falls prevention among older adults. *Int J Evid Based Healthc* 2015;**13**:213–23. doi:10.1097/XEB.0000000000000029

Figure legends:

Figure 1: Semi-structured interview questions for clinicians

Figure 2: Clinician preferences for the specific measurements that they would like Flexifoot to obtain

For peer review only

Contextual Background

- What is your age and nationality (you can prefer not to say)?
- What is/can you describe your professional role?
- How long have you been in this role?
- What types of patients do you come into contact with?
  A. Is there a particular type/category e.g OA/ACL?
  B. Does it vary?
  C. How many patients do you see per day?
  D. Is there a particular age range of patients you work with?

Wearable Technologies

- What do you know about wearable technologies?
- Do you currently use any wearable technologies in your work?

Clinician Preferences for the Flexifoot Device (With Reference to OA Surgery and ACL Injuries)

- Would you find Flexifoot useful for your OA and/or ACL injury patients?
- How would such a device help you in your own work? How would you use it?
- What would you specifically like to measure using the Flexifoot device?
- Would you like information about e.g.
  A. the patient's gait, such as symmetry/stride length?
  B. centre of pressure and pressure profiles for subsections of the foot?
  C. ground reaction force?
- Is there any parameters that should be tailored for OA surgery or for ACL injuries patients? (Different measurements used in accordance to the patients?)
- In addition to specific parameters, would you find it useful to measure the activity level of your patients? (e.g. time when active or not)
- How do you feel about monitoring compliance to exercise programmes?
- How often and for how long would you want the patient to wear the device?
- How would you like the data presented to you? E.g. in graphs, summary tables, performance profiles over a certain period of time?
- Would you find it useful to have a brief summary of the patient's progress with the option to look in more detail at certain aspects of the data?
- When would you like the data to be available to you? E.g. every day the patient uses Flexifoot?
- How would you ideally access the data? Would you like it emailed to you or would you prefer to have a website where you can log in to access it?
- Would you find an automatic alert system useful that told you when the data was available from your patient?
- Would you like an alert that will flag up if the metrics you identified fall below a certain threshold? (e.g. the patient is not doing exercise at all, so you need to send them a reminder?)
- Would you find Flexifoot to be more useful for patients with ACL injury or for pre-/post-OA surgery?
- What other information would you use to complement the use of Flexifoot in your clinical practice? Any other parameters that you think will be valuable that cannot be measured with Flexifoot?
- Can you suggest any reasons that may prevent you from using this device should it became available for use in clinical practice?

Closure

- Do you have any other comments about the Flexifoot device?

Semi-structured interview questions for clinicians

583x825mm (72 x 72 DPI)

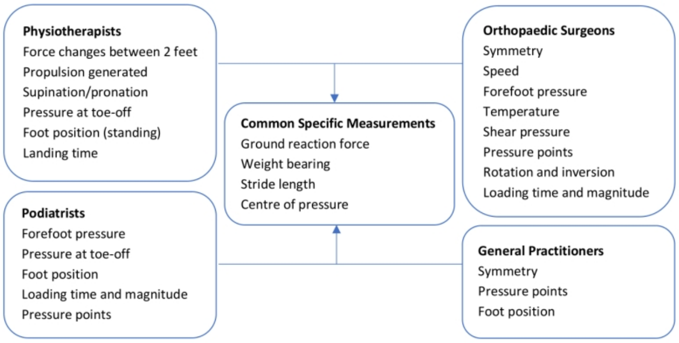

Clinician preferences for the specific measurements that they would like Flexifoot to obtain

583x825mm (72 x 72 DPI)

BMJ Open

Exploring the clinical context of adopting an instrumented insole: a qualitative study of clinicians' preferences

Journal:	*BMJ Open*
Manuscript ID	bmjopen-2018-023656.R1
Article Type:	Research
Date Submitted by the Author:	06-Dec-2018
Complete List of Authors:	Lin, Denise; Imperial College London Department of Surgery and Cancer<br>Papi, Enrica; Imperial College London, Department of Surgery and Cancer<br>McGregor, Alison; Imperial College London, Dept of Surgery and Cancer
**Primary Subject Heading**:	Qualitative research
Secondary Subject Heading:	Rehabilitation medicine
Keywords:	wearable technologies, Osteoarthritis, Self-managment, Pressure sensors, Users' preferences

SCHOLARONE™
Manuscripts

Exploring the clinical context of adopting an instrumented insole: a qualitative study of clinicians' preferences

Denise Lin[a], Enrica Papi[a,b], Alison McGregor[a]

[a]Department of Surgery and Cancer, Imperial College London, London, UK
[b] Department of Bioengineering, Imperial College London, London, UK

Corresponding author:
Dr Enrica Papi
Department of Surgery and Cancer,
Imperial College London,
Room 7L16, Floor 7, Laboratory Block,
Charing Cross Hospital,
London, W6 8RF, UK
Email: e.papi@imperial.ac.uk
Phone: 020 3313 8833

Denise Lin: email: denise.lin14@imperial.ac.uk

Alison H McGregor: email: a.mcgregor@imperial.ac.uk

Keywords: wearable technologies, osteoarthritis, self-management, users preferences, pressure sensors
Words Count: 4186

ABSTRACT

Objectives: This study explores clinicians' views of the clinical uptake of a smart pressure-sensing insole, named Flexifoot, to enhance the care and management of patients with osteoarthritis (OA). Clinicians are key users of wearable technologies, and can provide appropriate feedback for a specific device for successful clinical implementation.

Design: Qualitative study with in-depth, semi-structured interviews, analysed using inductive analysis to generate key themes.

Setting: Conducted in a University setting.

Participants: 30 clinicians were interviewed (11 physiotherapists, 11 orthopaedic surgeons, 5 general practitioners, 3 podiatrists).

Results: All clinicians regarded Flexifoot to be useful for the care and management of patients in adjunction to current methods. Responses revealed four main themes: use, data presentation, barriers to use, and future development. Flexifoot data was recognised as capable of enhancing information exchange between clinicians and patients, and also between clinicians themselves. Participants supported the use of the feedback for rehabilitation, screening and evaluation of treatment progress/success purposes. Flexifoot use by patients was encouraged as a self-management tool that may motivate them by setting attainment goals. The data interface should be secure, concise and visually appealing. The measured parameters of Flexifoot, its duration of wear and frequency of data output would all depend on the rationale for its use. The clinicians and patients must collaborate to optimise the use of Flexifoot for the long-term monitoring of disease for patient care in clinical practice. Many identified potential other uses for Flexifoot.

Conclusions: Flexifoot may complement and improve current methods of long-term patient management for OA or other conditions in clinical settings. The role of Flexifoot may be useful for objective measures and should be tailored carefully for each person and condition to maximise compliance. Adopting the device, and other similar technologies, requires reducing the main barriers to use (time, cost, patient compliance) before its successful implementation.

Strengths and limitations of this study

- This was the first qualitative study to specifically explore clinicians' views on implementing an in-house smart, flexible, pressure-sensing insole tool into clinical practice for patients with osteoarthritis
- The views of clinicians were fully explored with in-depth interviews, and analysed with inductive thematic analysis, giving rise to detailed suggestions to optimise the device's role alongside current strategies in patient care
- Clinicians were unable to use the device themselves prior to the interviews and responses were based upon one single demonstration and explanation of the tool
- Clinicians had a varied level of experience and familiarity of wearable technologies between them, influencing their perspectives

INTRODUCTION

Osteoarthritis (OA) is one of the most common long-term musculoskeletal diseases, and cause of pain and functional disability[1]. Individuals who have sustained a knee injury, such as anterior cruciate ligament (ACL) injuries, are 3-6 times more likely to develop knee OA[2–4]. In these patients, diagnosis occurs approximately 10 years prior to those without a previous injury[3,4], calling for long-term management of such conditions. Current clinical guidelines recommend physical activity to delay surgical intervention that is known to have a limited lifespan and in many instances poor reported patient outcomes[5–8], despite the belief that joint replacement is one of the most successful surgical procedures. Conversely, poor patient compliance limits long-term exercise benefits for OA, and many disregard the benefits of exercise.

Pain and gait changes are reasons why OA patients primarily visit clinicians. Gait analysis helps to establish OA diagnoses, severity and biomechanics underpinning musculoskeletal disorders[9]. In clinical settings, however, patient gait is observed by the clinician's eye, and self-reported questionnaires, such as the *36-Health Survey (SF-36)*, *Western Ontario and McMaster Universities Osteoarthritis Index (WOMAC)*, *Oxford Knee Score (OKN)* and *Knee Osteoarthritis Outcome Scores (KOOS)*, can assess OA severity[9–11]. Gait monitoring through clinician observation and patient questionnaires are prone to subjective responses, and therefore, are inadequate methods to quantify symptoms.

The emergence of wearable technologies can enhance current tools of physiotherapy, rehabilitation and daily monitoring of physical activity. Novel, portable wearable technologies offer a promising approach for use outside of the laboratory, to monitor functional changes in disease progression and activity levels. Nevertheless, the clinical implementation of wearable technologies is seemingly difficult. To enhance translation into clinical context, patient and clinicians' preferences have been explored in the past to determine the views and criteria of users for wearable technologies[12]. OA patients revealed that tracking disease progress was appealing and encouraged exercise[13]. Thirteen clinicians supported wearable technologies for OA patients clinically, but the preferences provided did not have specificity to one device[14].

Within our group, we developed a smart, flexible, pressure-sensing insole, aptly named 'Flexifoot' (Figure 1). Flexifoot generates plantar pressure readings from various foot regions. A high-resolution pressure map can be created from data that feeds back wirelessly to a smartphone app, for the extrapolation of gait spatio-temporal parameters, centre of pressure and pressure distribution. Flexifoot, being portable and low-cost to manufacture (approximately £20), in contrast to laboratory based-force-platforms, allows for continuous data collection over a substantial number of gait cycles, for feedback to patients and clinicians as needed. Daily gait and pressure analysis can enable patients to monitor improvements and disease-related progression, as well as guide clinicians through treatment decisions. Flexifoot is yet to be validated, therefore exploring users' preferences is beneficial for its development.

The ambiguity of previously obtained clinician preferences lacks the definitive feedback required to improve the design of a specific tool. The lack of specificity can be addressed by probing more into the

For peer review only - http://bmjopen.bmj.com/site/about/guidelines.xhtml

details of the clinical implementation of Flexifoot for OA and other disorders. The diagnosis and management of OA involves a multitude of healthcare professional types and it is therefore important to understand how Flexifoot could best address their requirements to inspire design and outcome measures which will facilitate clinical uptake. The aims of this study were to explore the clinicians' preferences for their use of Flexifoot and to identify specific parameters to be measured by the tool to foster improvements, and ultimately enhance OA patient care.

METHODS

Study Design

The study was a qualitative study based on in-depth semi-structured interviews with 30 clinicians. The study was reviewed and approved by Imperial College London Ethics Research Committee. All participants gave written informed consent prior to participation.

Participants

30 clinicians (18 males and 12 females, aged 21-57), including 11 physiotherapists, 11 orthopaedic surgeons, 5 general practitioners (GPs) and 3 podiatrists, were recruited for one-to-one interviews. The recruitment, via telephone and email, began from October 2015 and interviews occurred until September 2017 when data saturation occurred.

The healthcare professionals practiced amongst private and National Health Service (NHS) settings within London and Greater London, one in Hereford, one in Cheltenham and one in Liverpool. They had from 4 months up to 28 years of experience within current specialities.

Interviews and Data

The interviews were performed by researchers (DL and MG) in person, except for 2, which were conducted over the telephone due to scheduling constraints. The researchers did not have any personal relationships with the study participants, and the group had prior experience in conducting qualitative studies[13,14]. Face-to-face interviews were audio-recorded and transcribed afterwards.

Prior to each interview, participants' consent was obtained and researchers explained project aims, described Flexifoot and showed a prototype to each clinician (except in telephone interviews). Open-ended questions prompted clinicians to explore perspectives regarding the relevance of Flexifoot in clinical practice. The interview questions (Supplementary File 1) highlighted Flexifoot's clinical influence, specific measurements, data presentation preferences, and gave scope for feedback and improvement.

The interview verbatim transcriptions were analysed using inductive thematic analysis[15], without prior theoretical influences, whereby key findings were analysed and collated into early themes by DL and EP separately. DL and EP then checked each other's data and themes, ensuring consistency and the generation of recurrent key themes.

Patient and Public Involvement

While patients and users were not directly involved in the design of this study, this study arose from previous work where patients highlighted that their views were not considered in the design of novel wearable devices, thereby limiting uptake and translation[13,14]. This study directly focuses on care practitioners' preferences and requirements.

RESULTS

The semi-structured interviews opened with questions to determine healthcare backgrounds of clinicians and the relevance of wearable technologies within their profession. 24 out of 30 clinicians were aware of wearable technologies, and 4 used them for patients.

Inductive analysis[15] of interview responses revealed four main themes, with sub-themes: use (applications, specific measurements, duration of wear), data presentation (data access, visual presentation, frequency of data), barriers to use and future development. The former three themes

For peer review only

surfaced from specific interview questions asked. The latter was brought about after clinicians offered feedback as to how Flexifoot could be improved. The themes will be hereinafter described and verbatim quotes are indicated by: PT (physiotherapists), OS (orthopaedic surgeons), P (podiatrists) and GPs (general practitioners), followed by randomly assigned numbers.

Uses

Applications

The main uses of Flexifoot identified by clinicians are shown in Table 1.

Table 1: The main five applications of Flexifoot in clinical practice identified by 30 clinicians
**Main Applications of Flexifoot in Clinical Practice**
• Assessing efficacy of treatment (pre- and post-treatment)
• Monitoring disease progression
• Feedback for patients and other clinicians
• Monitoring activity levels and compliance
• Screening test to support future management

All groups of clinicians recognised Flexifoot as an objective outcome measure tool to monitor various parameters. 23 clinicians expressed that Flexifoot objective measures are useful to assess symptoms, and progress before and after medical intervention.

"It would be useful as an objective outcome measure for change…assess patients at time intervals for pre- and post-surgery." PT7

"This would be very interesting for research or pre- and post-surgery because you'd be able to monitor and look at change…it would definitely be useful as a follow-up guidance to surgical correction." OS11

21 clinicians felt that objective data can reinforce clinical interpretations and enhance information exchange between healthcare professionals. Moreover, real-time objective feedback can help to visually demonstrate the problem and solutions to patients.

"For us feeding back to the surgeons…you can be a bit more accurate about what it is that you're saying." PT11

"It might be useful to demonstrate to the patient what some of their symptoms are. To give them a visual representation of that, I could show them this while they're walking." OS10

"It's important for the patients to visualise what the problem is…as a relatively low-grade, without major intervention, you could do a lot with it to see how to correct problems objectively…It may then allow them to see visually what the issue is, so that if they correct it with the help of someone." GP2

Clinicians recognised Flexifoot as a self-management tool: rehabilitation targets can be set by patients themselves, or by clinicians, and motivate patients.

"For patients to use at home as a rehabilitation tool to set targets or goals they can monitor themselves." PT8

"Anything that can give feedback to the patient themselves, to become more active and more healthy, then that could be of benefit, not necessarily to me, but to the patient." OS9

75% of the clinicians supported Flexifoot measuring compliance to clinical advice.

"It would be really useful in terms of keeping a diary of what your patients are doing, especially with the osteoarthritis patients." PT5

"It is helpful if you have any doubts as to whether the patients are being compliant, if they are doing too much or too little activity." OS10

For peer review only

Clinicians can use the feedback as a screening tool, and to help determine the next steps for patient management.

"You could use that as some kind of screening test…do they really need to have a knee replacement yet?" OS1

"In conjunction with physiotherapists…so if you were trying to get them to do a particular rehabilitation programme. Monitor what they're doing, that might be very useful." OS6.

However, GPs felt that Flexifoot was a tool to be used more by patients, rather than by GPs for planning patient care: *"an intervention that's positive for the patient, as opposed to this being an investigation" GP2,* and that feedback would be better interpreted by clinicians with greater musculoskeletal knowledge.

"I can't see any acute use for it that's going to change the patient's management right this second now." GP2

"I'm not sure whether this would change my management for the conditions." GP5

Moreover, various participants in all clinician groups agreed that Flexifoot may be more effective not as a stand-alone device, but to enhance current methods for diagnosis and prognosis, as an *"adjunctive thing to what I already have" P3*.

Specific Measurements

Due to the vast array of parameters that can be analysed after Flexifoot use, it was important to determine the most clinically appropriate specific measurements. Participants were prompted by suggestions of symmetry, stride length, centre of pressure, pressure profiles or ground reaction force. The ideal specific measurements differed slightly between clinician groups due to varying levels of knowledge, but there was a convergence of agreed parameters to be measured by Flexifoot in all clinician groups (Figure 2). GPs provided fewer preferable specific measurements due to gaps in their specialist knowledge regarding musculoskeletal disorders.

The clinicians who supported the monitoring of activity levels (type and number of steps) demonstrated its importance for non-OA conditions too.

"It would give you an idea of their daily routine and exercise levels and things, particularly if they've got other conditions such as diabetes or cardiovascular disease which can be improved by exercise." GP3

Two clinicians exhibited a cautious view regarding tracking patients' activity outside of clinical environments:

"You've got to be careful with monitoring… you don't want engender this sort of 'we're watching you' big brother idea." PT3

"I do think that there could be some, a little degree of patients being suspicious of you checking up on them and they may question the clinician – why can't you trust what you're telling them, why do they need to see what I'm doing?" GP2.

Duration of Wear

The duration of Flexifoot wear suggested by clinicians varied from single usage at clinic appointments, up to long-term periods of over one year, implying a range of uses. The nature of the tool being able to offer real-time advice allows for its acute use in the clinic. The prolonged use of Flexifoot may be appropriate for patients with chronic diseases, or post-operatively in those undergoing surgical interventions. The disparity between preferred lengths depended on the rationale for patient use. 7 clinicians revealed that the duration of Flexifoot wear would be dependent on what outcomes were to be achieved (PT1, PT6, PT8, P2, OS1, OS6), type of injury (PT10), and patient age and compliance (GP5).

"If we were using it as a 'how do they move', we want a picture of their footprint, 5 minutes as they walk along the corridor." PT1

For peer review only

"During periods of activity, if they're experiencing pain or there is a particular challenging part of their day…short bursts of time which are critical to look at, so definitely not all day." OS3

However, long-term monitoring was preferred by most clinicians, where data collection would span over days, weeks, months and years, or *"for as long as it took to establish a meaningful change" PT3.*

"For 24hours or a few days…you'd want the results of this to reflect what they normally do." GP3

A short period of time using the device but at longer intervals between uses was also suggested:

"Snapshots at certain periods, much like we do at follow-up, at 6 weeks, 3 months, and a year." OS4

To maximise the personalised use of Flexifoot, clinicians and patients must collaborate to choose an appropriate duration of wear for each particular case.

Data Presentation

Data Access

50% of the clinicians would prefer to access the data by logging into a system, and some expressed that it should be integrated amongst patients' current notes for information crossover.

"If there was a way of tying in the results so that when you log in and click on the patient, it came up on their results. That would be the most ideal way." GP3

5 clinicians preferred the data via email, although 3 clinicians stated that maintaining patient confidentiality was paramount.

"Emailed is easier, but then log-in would be better because safety aspects and confidentiality." P2

Visual Presentation

The data should be concise so that clinicians can quickly interpret data. The clinicians expressed differing opinions after they were prompted with suggestions of graphs, tables and pictures. Therefore, numerous display options should be available to accommodate for all preferences.

"We need to have a summary that is brief, because you don't want to look through tons and tons of data. Then, if you wanted to look into more detail, then there should be the option." GP4

The data should be easily comprehended by patients too. A visually appealing approach with an additional colour-system scheme can enhance patient understanding.

"Patients want to know as well, they want a variation that's patient friendly…an easier format for patients to understand." OS6.

The addition of normal reference ranges alongside objective measures allows for the comparison of patient parameters versus reference data; this will enhance user-experiences and allow for goals to be set.

"Patient specific – it would be graphs. It would be nice to have a normal distribution and see where they fit inside the normal distribution." OS9

Frequency

The clinicians want to receive and access data at appropriate times, such as immediately before or during appointments. The majority (28/30) of clinicians were unlikely to monitor patients outside of the clinic due to time-restraints.

"I'd access the data just prior to the patient coming in or during that appointment. We would only really have time to monitor or review the data when the patient is actually here." PT5

"Real-time is helpful for the patient in a therapy session. If they had information once a week on how they're doing. Alerts are ideal, but there is no time to check it." PT10

For peer review only

The generation of data should occur in a timely fashion, *"correlated with the patient's clinic appointment" OS9.* The frequency of data received should depend on personal clinician preferences and the purpose of Flexifoot use.

"There should be an option for the data to be accessible when you want it and you choose." GP5

Barriers to Use

Clinicians highlighted obstacles to implementing Flexifoot (Table 2).

Table 2: The barriers to the use of implementing Flexifoot in clinical practice identified by 30 clinicians
**Barriers to the Use of Flexifoot**
• Time
• Cost and availability
• Influence on practice
• Training/education required
• Patient compliance
• Hygiene control

50% of the clinicians illustrated that time restraints were the largest concern of Flexifoot use clinically, including time taken for initial patient assessments, device introduction, and data generation.

"For me to use this for one patient, explain how to use it and monitor their activity is probably unlikely and unrealistic given the general practice workload and increasing demands on GPs." GP2

"That is the difficulty with this, it is an additional investigation that we need to spend time assessing." OS11

The second most identified barrier was cost – *"it has to be suitably priced…that an average practice can afford" P1.*

The inability of clinicians and patients to interpret data and the training required was another issue. 5 GPs stressed that Flexifoot was too specialised for their environment.

"Patients might not understand the data…people are not familiar with technologies, but this will be less of a problem in the future." PT9

"If it's mainly biometrics and gait analysis…I don't think that I would see this as being within a GP's remit so much. The biggest barrier for me not using it is, identifying how the information it gives would fall into my remit, and how it would influence my practice". GP3

Poor patient compliance also hinders Flexifoot's prospective use.

"Patients are so unreliable and I wouldn't be confident that they would remember to transfer it to another pair of shoes or if they take their shoes off and we'd not be tracking anything." OS3

6 clinicians identified hygiene and infection risks if the device was used for long periods, or between different patients. Flexifoot use between multiple patients could be more economically practical however.

"I suppose you've got questions of hygiene…you'd need a material you can wipe and maybe some way of cleaning them really well, so they would be to an infection control standard. If you can use them more than once, it would be cost effective." P3

Future Development

For peer review only

Following the responses to the set questions, clinicians offered suggestions on how to optimise Flexifoot for successful clinical uptake.

Parameters that would be ideally measured using Flexifoot (Figure 2) could be adapted to measure more factors and expand patient target audiences. Clinicians suggested Flexifoot use in Parkinson's disease, peripheral neuropathy in diabetes, chronic pain conditions, obesity, hemiplegia and tendinopathies.

"Pressure profiles are good, but it'd be really great to measure shear and temperatures in the foot…it would be better and more useful for people with plantar foot pain. Diabetics – it would be great." OS11

Design changes were also proposed, *"maybe this ribbon (ankle strap) could be a bit smaller because it might irritate someone on the side and it might artificially affect their gait" OS10.*

DISCUSSION

A technology must be user-friendly to optimise its efficacy and sustainability[16,17]. We conducted structured interviews with clinicians to guide the development of our novel technology, Flexifoot, towards clinical uptake. The clinicians expressed numerous advantages of adopting Flexifoot into healthcare settings and barriers, indicating strategies for future improvement. The main advantage recognised by clinicians was the ability of Flexifoot to generate quantitative data, that can be used for monitoring and feedback in various clinical contexts.

Clinicians implied that Flexifoot would not replace current clinical tools, but instead complement them. The ambulatory quantitative data can support existing OA diagnostic and management tools, and help to improve the reliability of clinical decisions. The ambulatory monitoring of disease progression, alongside patients' responses to treatment, has been considered useful[18]. Other rehabilitation technologies that motivate and offer objective feedback have been associated with long-term benefits and good physical fitness levels[19]. Moreover, objective feedback can enable a more efficient exchange and handover of patient information between clinicians[20]**.** A clinician may use the tool as a screening approach in adjunction to current methods, for more reliable results and subsequently refer the patient onto a specialist[14]. Also, the clinicians recognised that the data can reinforce their dialogue with patients, making patients more aware of their problem. Awareness and feedback was seen as a way to enhance patient self-management. Tracking activity levels and feedback engages patient involvement in their own care, and is useful for other non-musculoskeletal chronic conditions too[21,22]. Despite this, lower limb wearable technologies for post-stroke rehabilitation have shown limited efficacy in activity improvement in the past, but mainly due to poor research methodologies[23]. However, there is a still a demand for self-management of rehabilitation with feedback using shoe insole pressure sensors[24]. The contradictory results in the literature regarding wearable technologies calls for a study that explores the users' perspectives to maximise acceptance. Clinicians, in our study, indicated how Flexifoot could be a feedback tool for patients. The data being available to patients allows for greater independence in self-monitoring and feedback of their own diseases in familiar environments outside of clinics[18]. Home-based training and monitoring devices showed higher patient satisfaction compared to similar care within clinics[25]. Self-management can also reduce economic burdens as the technology can educate OA patients, improve outcomes and reduce hospital visits[18,26]. The uses and specific measurements suggested by clinicians was greatly dependent on the type of clinician and specific patient cohorts, which is also the case for other musculoskeletal interventions[27]. The GPs felt that they had insufficient gait analysis knowledge, and that the device was presently too specialist for general practice. GP environments may be inappropriate since it comprises of too broad a range of patients. Instead, they indicated that Flexifoot would be better suited for clinicians that follow up patients more regularly. This was reiterated by physiotherapists, orthopaedic surgeons and podiatrists, who are better equipped in musculoskeletal fields, and expressed positivity for Flexifoot's clinical uptake.

All clinicians indicated that the system and data output should be easy to use and interpret. Ease of use of wearable sensors for clinicians was reported in the past to facilitate their adoption into clinical settings[28]. The prospect of a log-in software system for Flexifoot data was more popular than receiving

For peer review only - http://bmjopen.bmj.com/site/about/guidelines.xhtml

results via email. The interface should be integrated alongside current patient records for information crossover and a choice of data presentation styles should be available. The material should be presented alongside normal reference ranges, for comparisons and targets to be made. Data accessibility and presentation should be understood by patients too – this is key for patient acceptance and accessibility[29]. Clinicians' views obtained in another study recognised that shorter, simpler and more concise data as educational material is preferable for patients, but that detailed data should be fully available too[30]. This agrees with our findings: participants expressed the possibility of having access to more detailed data if needed, whilst avoiding scanning excessive data beyond their understanding. Full data measurements could be stored however for more skilled users in research settings[31].

Furthermore, shortcomings of Flexifoot were recognised by the clinicians. The clinicians' continuous workloads means that using real-time data from Flexifoot is only feasible prior to or during appointments in the presence of patients. The real-time data and automatic alerts may be more useful for patient users for receiving feedback, which continues to motivate them[13]. Clinicians are reluctant for the introduction of new tools because they can disrupt time-pressured practice schedules, and the time required to train them to use Flexifoot must also be considered[32]. The clinical efficacy of Flexifoot must be therefore fully established, such as through patient usability testing, before clinicians can adopt it as a method worthy of appointment time. High costs also limit new technology implementation within health services. The current expense to manufacture one Flexifoot device is low, but one tool per patient may be economically impractical. The recycling and reuse of devices between patients may reduce costs, but increases hygiene and infection risks, expressed by 6 clinicians. Introducing hygienic procedures prior to and after Flexifoot use could enhance its reusability and cost-effectiveness. Thus, the cost-effectiveness, with reference to current treatment guidelines, should be further investigated. Patient compliance is also an issue since long-term wearable devices require adequate patient acceptability. However, from a similar study we conducted that explored patients' views, all participants were keen for the uptake of wearable technologies[13]. Moreover, effective clinician-patient communication can determine patient cohorts supportive of self-management, and those more likely to adhere to using Flexifoot[13,14,33]. Clinicians can promote the relevance of sensor technologies for patients' care, and hence boost compliance[12,34]. The identified issues surrounding Flexifoot are apparent in other wearable technologies too[27]. The replication of problems between devices implies a necessity for new approaches in encouraging patients' compliance and appeal for novel strategies. The findings that emerged from this study can be translated to other similar technologies to promote their clinical uptake and foster new developments.

The study limitations involved the clinicians' varied levels of experience and familiarity of instrumented insoles, and that 27/30 interviewees were based in the London area. The clinicians had not used Flexifoot, and telephone interviews could not view the device, but detailed descriptions and commercially similar devices that could be found online were provided prior to interviews. Future studies would involve clinicians' use of Flexifoot beforehand. Moreover, clinicians who were well-informed around the subject were perhaps biased to initially participate and express positivity. However, the interviews were confidential and honest feedback was encouraged.

In conclusion, the clinicians considered Flexifoot to be a useful tool that could be used in adjunction to current approaches, in a long-term, follow-up setting to support and improve patient care. The clinicians' preferences exhibited numerous ways in which Flexifoot can be useful for patients with OA or other conditions. The measured parameters should be selected according to patient-specific cases, and delivered in a concise manner through a secure interface. A choice of data outputs should be offered to cater for all users. The challenges of time, cost, infection control should be addressed, alongside the clinical efficacy and cost-effectiveness for the clinical adoption of Flexifoot and similar technologies.

Author Contributions: DL, EP, and AHM conceived and designed the study. DL carried out the interviews; DL and EP analysed and interpreted the data. DL and EP drafted the manuscript. All authors read, edited,

For peer review only

and approved the final version of the manuscript. All authors had full access to all of the data in the study and can take responsibility for the integrity of the data and the accuracy of the data analysis.

Acknowledgement: We would like to thank MSc student Mary Goodwin for her contribution with data collection.

Funding: This research received no specific grant from any funding agency in the public, commercial or not-for-profit sectors

Competing interests: The authors declare that there are no conflicts of interest.

All authors have completed the ICMJE uniform disclosure form at www.icmje.org/coi_disclosure.pdf (available on request from the corresponding author) and declare: no support from any organisation for the submitted work; no financial relationships with any organisations that might have an interest in the submitted work in the previous three years; no other relationships or activities that could appear to have influenced the submitted work.

Ethical approval: The study was reviewed and approved by Imperial College London Ethics Research Committee. All participants gave written informed consent.

Data sharing: No additional data available.

For peer review only

References

1 Bennell KL, Hunter DJ, Hinman RS. Management of osteoarthritis of the knee. *Bmj* 2012;**345**:e4934–e4934. doi:10.1136/bmj.e4934

2 Simon D, Mascarenhas R, Saltzman BM, *et al.* The Relationship between Anterior Cruciate Ligament Injury and Osteoarthritis of the Knee. *Adv Orthop* 2015;**2015**. doi:10.1155/2015/928301

3 Muthuri SG, McWilliams DF, Doherty M, *et al.* History of knee injuries and knee osteoarthritis: A meta-analysis of observational studies. *Osteoarthr Cartil* 2011;**19**:1286–93. doi:10.1016/j.joca.2011.07.015

4 Driban JB, Eaton CB, Lo GH, *et al.* Association of Knee Injuries With Accelerated Knee Osteoarthritis Progression: Data From the Osteoarthritis Initiative. *Arthritis Care Res* 2014;**66**:1673–9. doi:10.1002/acr.22359

5 Jones CA, Voaklander DC, Johnston DW, *et al.* Health related quality of life outcomes after total hip and knee arthroplasties in a community based population. *J Rheumatol* 2000;**27**:1745–52.http://www.ncbi.nlm.nih.gov/pubmed/10914862 (accessed 29 Mar 2018).

6 Wylde V, Hewlett S, Learmonth ID, *et al.* Persistent pain after joint replacement: Prevalence, sensory qualities, and postoperative determinants. *Pain* 2011;**152**:566–72. doi:10.1016/j.pain.2010.11.023

7 Singh JA, O'Byrne M, Harmsen S, *et al.* Predictors of moderate-severe functional limitation after primary Total Knee Arthroplasty (TKA): 4701 TKAs at 2-years and 2935 TKAs at 5-years. *Osteoarthr Cartil* 2010;**18**:515–21. doi:10.1016/j.joca.2009.12.001

8 Dowsey MM, Nikpour M, Dieppe P, *et al.* Associations between pre-operative radiographic changes and outcomes after total knee joint replacement for osteoarthritis. *Osteoarthr Cartil* 2012;**20**:1095–102. doi:10.1016/j.joca.2012.05.015

9 Elbaz A, Mor A, Segal G, *et al.* Novel classification of knee osteoarthritis severity based on spatiotemporal gait analysis. *Osteoarthr Cartil* 2014;**22**:457–63. doi:10.1016/j.joca.2013.12.015

10 Harris KK, Dawson J, Jones LD, *et al.* Extending the use of PROMs in the NHS—using the Oxford Knee Score in patients undergoing non-operative management for knee osteoarthritis: a validation study. *BMJ Open* 2013;**3**:e003365. doi:10.1136/bmjopen-2013-003365

11 Roos EM, Roos HP, Lohmander LS, *et al.* Knee Injury and Osteoarthritis Outcome Score (KOOS)—Development of a Self-Administered Outcome Measure. *J Orthop Sport Phys Ther* 1998;**28**:88–96. doi:10.2519/jospt.1998.28.2.88

12 Bergmann JHM, McGregor AH. Body-worn sensor design: What do patients and clinicians want? *Ann Biomed Eng* 2011;**39**:2299–312. doi:10.1007/s10439-011-0339-9

13 Papi E, Belsi A, McGregor AH. A knee monitoring device and the preferences of patients living with osteoarthritis: a qualitative study. *BMJ Open* 2015;**5**:e007980. doi:10.1136/bmjopen-2015-007980

14 Papi E, Murtagh GM, McGregor AH. Wearable technologies in osteoarthritis: a qualitative study of

clinicians' preferences. *BMJ Open* 2016;**6**:e009544. doi:10.1136/bmjopen-2015-009544
15 Ritchie J, Lewis J. QUALITATIVE RESEARCH PRACTICE A Guide for Social Science Students and Researchers. https://mthoyibi.files.wordpress.com/2011/10/qualitative-research-practice_a-guide-for-social-science-students-and-researchers_jane-ritchie-and-jane-lewis-eds_20031.pdf (accessed 13 Dec 2017).
16 Lewis JE, Neider MB. Designing Wearable Technology for an Aging Population. *Ergon Des* 2017;**25**:4–10. doi:10.1177/1064804616645488
17 Lee J, Kim D, Ryoo H-Y, *et al.* Sustainable Wearables: Wearable Technology for Enhancing the Quality of Human Life. *Sustainability* 2016;**8**:466. doi:10.3390/su8050466
18 Majumder S, Mondal T, Deen M. Wearable Sensors for Remote Health Monitoring. *Sensors* 2017;**17**:130. doi:10.3390/s17010130
19 Aamot I-L, Karlsen T, Dalen H, *et al.* Long-term Exercise Adherence After High-intensity Interval Training in Cardiac Rehabilitation: A Randomized Study. *Physiother Res Int* 2016;**21**:54–64. doi:10.1002/pri.1619
20 Popovici I, Morita PP, Doran D, *et al.* Technological aspects of hospital communication challenges: An observational study. *Int J Qual Heal Care* 2015;**27**:183–8. doi:10.1093/intqhc/mzv016
21 Chiauzzi E, Rodarte C, DasMahapatra P. Patient-centered activity monitoring in the self-management of chronic health conditions. *BMC Med* 2015;**13**:1–6. doi:10.1186/s12916-015-0319-2
22 Thornton JS, Frémont P, Khan K, *et al.* Physical activity prescription: a critical opportunity to address a modifiable risk factor for the prevention and management of chronic disease: a position statement by the Canadian Academy of Sport and Exercise Medicine. *Br J Sports Med* 2016;**50**:1109–14. doi:10.1136/bjsports-2016-096291
23 Powell L, Parker J, Martyn St-James M, *et al.* The Effectiveness of Lower-Limb Wearable Technology for Improving Activity and Participation in Adult Stroke Survivors: A Systematic Review. *J Med Internet Res* 2016;**18**:e259. doi:10.2196/jmir.5891
24 Munoz-Organero M, Parker J, Powell L, *et al.* Assessing Walking Strategies Using Insole Pressure Sensors for Stroke Survivors. *Sensors (Basel)* 2016;**16**. doi:10.3390/s16101631
25 Kraal JJ, Elske Van Den Akker-Van Marle M, Abu-Hanna A, *et al.* Clinical and cost-effectiveness of home-based cardiac rehabilitation compared to conventional, centre-based cardiac rehabilitation: Results of the FIT@Home study. *Eur J Prev Cardiol* 2017;**24**:1260–73. doi:10.1177/2047487317710803
26 Bodenheimer T, Lorig K. Patient Self-management of Chronic Disease in Primary Care. 2014;**288**:2469–75. doi:10.1001/jama.288.19.2469
27 de Almeida PHTQ, MacDermid JC, Pontes TB, *et al.* Orthotic use for CMC osteoarthritis: Variations among different health professionals in Brazil. *J Hand Ther* 2016;**29**:440–50. doi:10.1016/j.jht.2016.07.007
28 Grimm B, Bolink S. Evaluating physical function and activity in the elderly patient using wearable motion sensors. *EFORT Open Rev* 2016;**1**:112–20. doi:10.1302/2058-5241.1.160022
29 Puri A, Kim B, Nguyen O, *et al.* User Acceptance of Wrist-Worn Activity Trackers Among Community-Dwelling Older Adults: Mixed Method Study. *JMIR mHealth uHealth* 2017;**5**:e173. doi:10.2196/mhealth.8211
30 Grudniewicz A, Bhattacharyya O, McKibbon KA, *et al.* User-centered design and printed educational materials: A focus group study of primary care physician preferences. *J Contin Educ Health Prof* 2016;**36**:249–55. doi:10.1097/CEH.0000000000000112
31 Iqbal MH, Aydin A, Brunckhorst O, *et al.* A review of wearable technology in medicine. *J R Soc Med* 2016;**109**:372–80. doi:10.1177/0141076816663560
32 Yarbrough a. K, Smith TB. A new take on TAM. *Med Care Res Rev* 2007;**64**:650–72. doi:10.1177/1077558707305942
33 Loos JR, Davidson EJ. Wearable health monitors and physician-patient communication: The physician's perspective. *Proc Annu Hawaii Int Conf Syst Sci* 2016;**2016–March**:3389–98. doi:10.1109/HICSS.2016.422
34 Teh RC-A, Mahajan N, Visvanathan R, *et al.* Clinical effectiveness of and attitudes and beliefs of health professionals towards the use of health technology in falls prevention among older adults. *Int J Evid Based Healthc* 2015;**13**:213–23. doi:10.1097/XEB.0000000000000029

For peer review only

Supplementary files: Semi-structured interview questions for clinicians

Figure legends:

Figure 1: a) Layout of pressure sensors on the insoles with connectors for the circuit boards. b) Insoles covered with neoprene with circuit boards for data transmission attached and ready to be inserted into shoes.

Figure 2: Clinician preferences for the specific measurements that they would like Flexifoot to obtain.

For peer review only

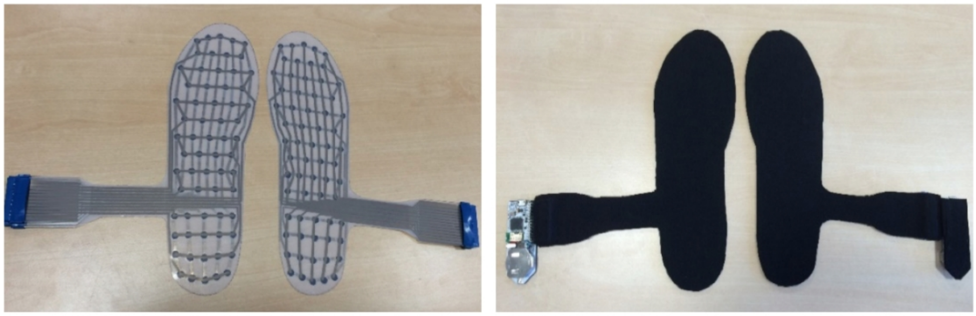

a b

Figure 1: a) Layout of pressure sensors on the insoles with connectors for the circuit boards. b) Insoles covered with neoprene with circuit boards for data transmission attached and ready to be inserted into shoes.

157x62mm (200 x 200 DPI)

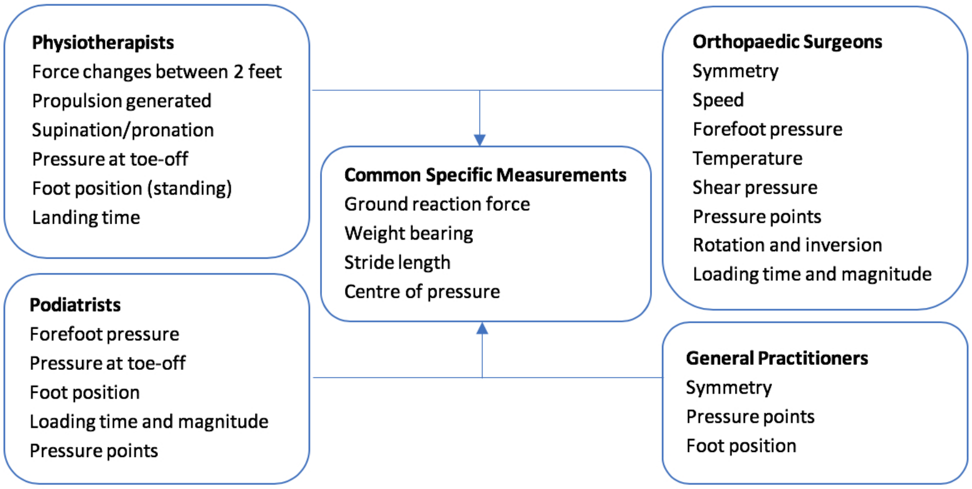

Figure 2: Clinician preferences for the specific measurements that they would like Flexifoot to obtain.

152x74mm (200 x 200 DPI)

Semi-structured interview questions for clinicians

Contextual Background

- What is your age and nationality (you can prefer not to say)?
- What is/can you describe your professional role?
- How long have you been in this role?
- What types of patients do you come into contact with?
  A. Is there a particular type/category e.g OA/ACL?
  B. Does it vary?
  C. How many patients do you see per day?
  D. Is there a particular age range of patients you work with?

Wearable Technologies

- What do you know about wearable technologies?
- Do you currently use any wearable technologies in your work?

Clinician Preferences for the Flexifoot Device (With Reference to OA Surgery and ACL Injuries)

- Would you find Flexifoot useful for your OA and/or ACL injury patients?
- How would such a device help you in your own work? How would you use it?
- What would you specifically like to measure using the Flexifoot device?
- Would you like information about e.g.
  A. the patient's gait, such as symmetry/stride length?
  B. centre of pressure and pressure profiles for subsections of the foot?
  C. ground reaction force?
- Is there any parameters that should be tailored for OA surgery or for ACL injuries patients? (Different measurements used in accordance to the patients?)
- In addition to specific parameters, would you find it useful to measure the activity level of your patients? (e.g. time when active or not)
- How do you feel about monitoring compliance to exercise programmes?
- How often and for how long would you want the patient to wear the device?
- How would you like the data presented to you? E.g. in graphs, summary tables, performance profiles over a certain period of time?
- Would you find it useful to have a brief summary of the patient's progress with the option to look in more detail at certain aspects of the data?
- When would you like the data to be available to you? E.g. every day the patient uses Flexifoot?
- How would you ideally access the data? Would you like it emailed to you or would you prefer to have a website where you can log in to access it?
- Would you find an automatic alert system useful that told you when the data was available from your patient?
- Would you like an alert that will flag up if the metrics you identified fall below a certain threshold? (e.g. the patient is not doing exercise at all, so you need to send them a reminder?)
- Would you find Flexifoot to be more useful for patients with ACL injury or for pre-/post-OA surgery?
- What other information would you use to complement the use of Flexifoot in your clinical practice? Any other parameters that you think will be valuable that cannot be measured with Flexifoot?
- Can you suggest any reasons that may prevent you from using this device should it became available for use in clinical practice?

Closure

- Do you have any other comments about the Flexifoot device?

For peer review only

Standards for Reporting Qualitative Research (SRQR)*

http://www.equator-network.org/reporting-guidelines/srqr/

Page/line no(s).

Title and abstract

**Title** - Concise description of the nature and topic of the study Identifying the study as qualitative or indicating the approach (e.g., ethnography, grounded theory) or data collection methods (e.g., interview, focus group) is recommended	Page 1: Lines 1-2
**Abstract** - Summary of key elements of the study using the abstract format of the intended publication; typically includes background, purpose, methods, results, and conclusions	Page 1: Lines 25-50

Introduction

**Problem formulation** - Description and significance of the problem/phenomenon studied; review of relevant theory and empirical work; problem statement	Page 2: Lines 15-51
**Purpose or research question** - Purpose of the study and specific objectives or questions	Page 2: Lines 49-54<br>Page 3: Lines 1-6

Methods

**Qualitative approach and research paradigm** - Qualitative approach (e.g., ethnography, grounded theory, case study, phenomenology, narrative research) and guiding theory if appropriate; identifying the research paradigm (e.g., postpositivist, constructivist/ interpretivist) is also recommended; rationale**	Page 3: Line 10-19,<br>Page 3: Lines 5-24-43
**Researcher characteristics and reflexivity** - Researchers' characteristics that may influence the research, including personal attributes, qualifications/experience, relationship with participants, assumptions, and/or presuppositions; potential or actual interaction between researchers' characteristics and the research questions, approach, methods, results, and/or transferability	Page 3: Lines 25-28<br>Page 10: Lines 5-6
**Context** - Setting/site and salient contextual factors; rationale**	Page 3: Lines 11-12, 16-22, 40-43
**Sampling strategy** - How and why research participants, documents, or events were selected; criteria for deciding when no further sampling was necessary (e.g., sampling saturation); rationale**	Page 2: Lines 53-54<br>Page 3: Lines 1-4, 40-43
**Ethical issues pertaining to human subjects** - Documentation of approval by an appropriate ethics review board and participant consent, or explanation for lack thereof; other confidentiality and data security issues	Page 3: Lines 11-13, 40-43
**Data collection methods** - Types of data collected; details of data collection procedures including (as appropriate) start and stop dates of data collection and analysis, iterative process, triangulation of sources/methods, and modification of procedures in response to evolving study findings; rationale**	Page 3: Line 11, 24-37

For peer review only - http://bmjopen.bmj.com/site/about/guidelines.xhtml

**Data collection instruments and technologies** - Description of instruments (e.g., interview guides, questionnaires) and devices (e.g., audio recorders) used for data collection; if/how the instrument(s) changed over the course of the study	Page 3: Lines 11, 24-37
**Units of study** - Number and relevant characteristics of participants, documents, or events included in the study; level of participation (could be reported in results)	Page 3: Lines 15-33, 46-48
**Data processing** - Methods for processing data prior to and during analysis, including transcription, data entry, data management and security, verification of data integrity, data coding, and anonymization/de-identification of excerpts	Page 3: Line 11, 24-37
**Data analysis** - Process by which inferences, themes, etc., were identified and developed, including the researchers involved in data analysis; usually references a specific paradigm or approach; rationale**	Page 3: Lines 24-37, 46-51<br>Page 4: 1-2
**Techniques to enhance trustworthiness** - Techniques to enhance trustworthiness and credibility of data analysis (e.g., member checking, audit trail, triangulation); rationale**	Page 3: Lines 34-37

Results/findings

**Synthesis and interpretation** - Main findings (e.g., interpretations, inferences, and themes); might include development of a theory or model, or integration with prior research or theory	Page 3: Lines 46-51<br>Page 4: Lines 1-3
**Links to empirical data** - Evidence (e.g., quotes, field notes, text excerpts, photographs) to substantiate analytic findings	Page 4: Lines 6-42<br>Pages 5-7<br>Page 8: Lines 1-10

Discussion

**Integration with prior work, implications, transferability, and contribution(s) to the field -** Short summary of main findings; explanation of how findings and conclusions connect to, support, elaborate on, or challenge conclusions of earlier scholarship; discussion of scope of application/generalizability; identification of unique contribution(s) to scholarship in a discipline or field	Page 8: Lines 13-52<br>Page 9: Lines 1-50
**Limitations** - Trustworthiness and limitations of findings	Page 9: Lines 35-41

Other

**Conflicts of interest** - Potential sources of influence or perceived influence on study conduct and conclusions; how these were managed	Page 10: Lines 7-12
**Funding** - Sources of funding and other support; role of funders in data collection, interpretation, and reporting	Page 10: Lines 5-6

For peer review only - http://bmjopen.bmj.com/site/about/guidelines.xhtml

*The authors created the SRQR by searching the literature to identify guidelines, reporting standards, and critical appraisal criteria for qualitative research; reviewing the reference lists of retrieved sources; and contacting experts to gain feedback. The SRQR aims to improve the transparency of all aspects of qualitative research by providing clear standards for reporting qualitative research.

**The rationale should briefly discuss the justification for choosing that theory, approach, method, or technique rather than other options available, the assumptions and limitations implicit in those choices, and how those choices influence study conclusions and transferability. As appropriate, the rationale for several items might be discussed together.

Reference:

O'Brien BC, Harris IB, Beckman TJ, Reed DA, Cook DA. **Standards for reporting qualitative research: a synthesis of recommendations.** *Academic Medicine*, Vol. 89, No. 9 / Sept 2014 DOI: 10.1097/ACM.0000000000000388

For peer review only

BMJ Open

Exploring the clinical context of adopting an instrumented insole: a qualitative study of clinicians' preferences in England

Journal:	*BMJ Open*
Manuscript ID	bmjopen-2018-023656.R2
Article Type:	Research
Date Submitted by the Author:	15-Jan-2019
Complete List of Authors:	Lin, Denise; Imperial College London Department of Surgery and Cancer<br>Papi, Enrica; Imperial College London, Department of Surgery and Cancer<br>McGregor, Alison; Imperial College London, Dept of Surgery and Cancer
**Primary Subject Heading**:	Qualitative research
Secondary Subject Heading:	Rehabilitation medicine
Keywords:	wearable technologies, Osteoarthritis, Self-managment, Pressure sensors, Users' preferences

SCHOLARONE™
Manuscripts

Exploring the clinical context of adopting an instrumented insole: a qualitative study of clinicians' preferences in England

Denise Lin[a], Enrica Papi[a,b], Alison McGregor[a]

[a]Department of Surgery and Cancer, Imperial College London, London, UK
[b] Department of Bioengineering, Imperial College London, London, UK

Corresponding author:
Dr Enrica Papi
Department of Surgery and Cancer,
Imperial College London,
Room 7L16, Floor 7, Laboratory Block,
Charing Cross Hospital,
London, W6 8RF, UK
Email: e.papi@imperial.ac.uk
Phone: 020 3313 8833

Denise Lin: email: denise.lin14@imperial.ac.uk

Alison H McGregor: email: a.mcgregor@imperial.ac.uk

Keywords: wearable technologies, osteoarthritis, self-management, users preferences, pressure sensors
Words Count: 4186

ABSTRACT

Objectives: This study explores clinicians' views of the clinical uptake of a smart pressure-sensing insole, named Flexifoot, to enhance the care and management of patients with osteoarthritis (OA). Clinicians are key users of wearable technologies, and can provide appropriate feedback for a specific device for successful clinical implementation.

Design: Qualitative study with in-depth, semi-structured interviews, analysed using inductive analysis to generate key themes.

Setting: Conducted in a University setting.

Participants: 30 clinicians were interviewed (11 physiotherapists, 11 orthopaedic surgeons, 5 general practitioners, 3 podiatrists).

Results: All clinicians regarded Flexifoot to be useful for the care and management of patients in adjunction to current methods. Responses revealed four main themes: use, data presentation, barriers to use, and future development. Flexifoot data was recognised as capable of enhancing information exchange between clinicians and patients, and also between clinicians themselves. Participants supported the use of the feedback for rehabilitation, screening and evaluation of treatment progress/success purposes. Flexifoot use by patients was encouraged as a self-management tool that may motivate them by setting attainment goals. The data interface should be secure, concise and visually appealing. The measured parameters of Flexifoot, its duration of wear and frequency of data output would all depend on the rationale for its use. The clinicians and patients must collaborate to optimise the use of Flexifoot for the long-term monitoring of disease for patient care in clinical practice. Many identified potential other uses for Flexifoot.

Conclusions: Flexifoot may complement and improve current methods of long-term patient management for OA or other conditions in clinical settings. The role of Flexifoot may be useful for objective measures and should be tailored carefully for each person and condition to maximise compliance. Adopting the device, and other similar technologies, requires reducing the main barriers to use (time, cost, patient compliance) before its successful implementation.

Strengths and limitations of this study

- This was the first qualitative study to specifically explore clinicians' views on implementing an in-house smart, flexible, pressure-sensing insole tool into clinical practice for patients with osteoarthritis
- The views of clinicians were fully explored with in-depth interviews, and analysed with inductive thematic analysis, giving rise to detailed suggestions to optimise the device's role alongside current strategies in patient care
- Clinicians were unable to use the device themselves prior to the interviews and responses were based upon one single demonstration and explanation of the tool
- Clinicians had a varied level of experience and familiarity of wearable technologies between them, influencing their perspectives

INTRODUCTION

Osteoarthritis (OA) is one of the most common long-term musculoskeletal diseases, and cause of pain and functional disability[1]. Individuals who have sustained a knee injury, such as anterior cruciate ligament (ACL) injuries, are 3-6 times more likely to develop knee OA[2–4]. In these patients, diagnosis occurs approximately 10 years prior to those without a previous injury[3,4], calling for long-term management of such conditions. Current clinical guidelines recommend physical activity to delay surgical intervention that is known to have a limited lifespan and in many instances poor reported patient outcomes[5–8], despite the belief that joint replacement is one of the most successful surgical procedures. Conversely, poor patient compliance limits long-term exercise benefits for OA, and many disregard the benefits of exercise.

Pain and gait changes are reasons why OA patients primarily visit clinicians. Gait analysis helps to establish OA diagnoses, severity and biomechanics underpinning musculoskeletal disorders[9]. In clinical settings, however, patient gait is observed by the clinician's eye, and self-reported questionnaires, such as the *36-Health Survey (SF-36)*, *Western Ontario and McMaster Universities Osteoarthritis Index (WOMAC)*, *Oxford Knee Score (OKN)* and *Knee Osteoarthritis Outcome Scores (KOOS)*, can assess OA severity[9–11]. Gait monitoring through clinician observation and patient questionnaires are prone to subjective responses, and therefore, are inadequate methods to quantify symptoms.

The emergence of wearable technologies can enhance current tools of physiotherapy, rehabilitation and daily monitoring of physical activity. Novel, portable wearable technologies offer a promising approach for use outside of the laboratory, to monitor functional changes in disease progression and activity levels. Nevertheless, the clinical implementation of wearable technologies is seemingly difficult. To enhance translation into clinical context, patient and clinicians' preferences have been explored in the past to determine the views and criteria of users for wearable technologies[12]. OA patients revealed that tracking disease progress was appealing and encouraged exercise[13]. Thirteen clinicians supported wearable technologies for OA patients clinically, but the preferences provided did not have specificity to one device[14].

Within our group, we developed a smart, flexible, pressure-sensing insole, aptly named 'Flexifoot' (Figure 1). Flexifoot generates plantar pressure readings from various foot regions. A high-resolution pressure map can be created from data that feeds back wirelessly to a smartphone app, for the extrapolation of gait spatio-temporal parameters, centre of pressure and pressure distribution. Flexifoot, being portable and low-cost to manufacture (approximately £20), in contrast to laboratory based-force-platforms, allows for continuous data collection over a substantial number of gait cycles, for feedback to patients and clinicians as needed. Daily gait and pressure analysis can enable patients to monitor improvements and disease-related progression, as well as guide clinicians through treatment decisions. Flexifoot is yet to be validated, therefore exploring users' preferences is beneficial for its ongoing development.

The ambiguity of previously obtained clinician preferences lacks the definitive feedback required to improve the design of a specific tool. The lack of specificity can be addressed by probing more into the details of the clinical implementation of Flexifoot for OA and other disorders. The diagnosis and

For peer review only

management of OA involves a multitude of healthcare professional types and it is therefore important to understand how Flexifoot could best address their requirements to inspire design and outcome measures which will facilitate clinical uptake. The aims of this study were to explore the clinicians' preferences for their use of Flexifoot and to identify specific parameters to be measured by the tool to foster improvements, and ultimately enhance OA patient care.

METHODS

Study Design

The study was a qualitative study based on in-depth semi-structured interviews with 30 clinicians. The study was reviewed and approved by Imperial College London Ethics Research Committee. All participants gave written informed consent prior to participation.

Participants

30 clinicians (18 males and 12 females, aged 21-57), including 11 physiotherapists, 11 orthopaedic surgeons, 5 general practitioners (GPs) and 3 podiatrists, were recruited for one-to-one interviews.

Clinicians that had previously or currently worked for the Imperial College Healthcare National Health Service (NHS) Trust were invited via telephone and email invitations to partake in our study. The recruitment began from October 2015 and interviews occurred until September 2017, when data saturation occurred.

At the time of clinician interviewing, the healthcare professionals practiced amongst private and NHS settings within London and Greater London, one in Hereford, one in Cheltenham and one in Liverpool. They had from 4 months up to 28 years of experience within current specialities.

Interviews and Data

The interviews were performed by researchers (DL and MG) in person, except for 2, which were conducted over the telephone due to scheduling constraints. The researchers did not have any personal relationships with the study participants, and the group had prior experience in conducting qualitative studies[13,14]. Face-to-face interviews were audio-recorded and transcribed afterwards.

Prior to each interview, participants' consent was obtained and researchers explained project aims, described Flexifoot and showed a prototype to each clinician (except in telephone interviews). Open-ended questions prompted clinicians to explore perspectives regarding the relevance of Flexifoot in clinical practice. The interview questions (Supplementary File 1) highlighted Flexifoot's clinical influence, specific measurements, data presentation preferences, and gave scope for feedback and improvement.

The interview verbatim transcriptions were analysed using inductive thematic analysis[15], without prior theoretical influences, whereby key findings were analysed and collated into early themes by DL and EP separately. DL and EP then checked each other's data and themes, ensuring consistency and the generation of recurrent key themes.

Patient and Public Involvement

While patients and users were not directly involved in the design of this study, this study arose from previous work where patients highlighted that their views were not considered in the design of novel wearable devices, thereby limiting uptake and translation[13,14]. This study directly focuses on care practitioners' preferences and requirements.

RESULTS

The semi-structured interviews opened with questions to determine healthcare backgrounds of clinicians and the relevance of wearable technologies within their profession. 24 out of 30 clinicians were aware of wearable technologies, and 4 used them for patients.

Inductive analysis[15] of interview responses revealed four main themes, with sub-themes: use (applications, specific measurements, duration of wear), data presentation (data access, visual

For peer review only

presentation, frequency of data), barriers to use and future development. The former three themes surfaced from specific interview questions asked. The latter was brought about after clinicians offered feedback as to how Flexifoot could be improved. The themes will be hereinafter described and verbatim quotes are indicated by: PT (physiotherapists), OS (orthopaedic surgeons), P (podiatrists) and GPs (general practitioners), followed by randomly assigned numbers.

Uses

Applications

The main uses of Flexifoot identified by clinicians are shown in Table 1.

Table 1: The main five applications of Flexifoot in clinical practice identified by 30 clinicians

**Main Applications of Flexifoot in Clinical Practice**
• Assessing efficacy of treatment (pre- and post-treatment)
• Monitoring disease progression
• Feedback for patients and other clinicians
• Monitoring activity levels and compliance
• Screening test to support future management

All groups of clinicians recognised Flexifoot as an objective outcome measure tool to monitor various parameters. 23 clinicians expressed that Flexifoot objective measures are useful to assess symptoms, and progress before and after medical intervention.

"It would be useful as an objective outcome measure for change…assess patients at time intervals for pre- and post-surgery." PT7

"This would be very interesting for research or pre- and post-surgery because you'd be able to monitor and look at change…it would definitely be useful as a follow-up guidance to surgical correction." OS11

21 clinicians felt that objective data can reinforce clinical interpretations and enhance information exchange between healthcare professionals. Moreover, real-time objective feedback can help to visually demonstrate the problem and solutions to patients.

"For us feeding back to the surgeons…you can be a bit more accurate about what it is that you're saying." PT11

"It might be useful to demonstrate to the patient what some of their symptoms are. To give them a visual representation of that, I could show them this while they're walking." OS10

"It's important for the patients to visualise what the problem is…as a relatively low-grade, without major intervention, you could do a lot with it to see how to correct problems objectively…It may then allow them to see visually what the issue is, so that if they correct it with the help of someone." GP2

Clinicians recognised Flexifoot as a self-management tool: rehabilitation targets can be set by patients themselves, or by clinicians, and motivate patients.

"For patients to use at home as a rehabilitation tool to set targets or goals they can monitor themselves." PT8

"Anything that can give feedback to the patient themselves, to become more active and more healthy, then that could be of benefit, not necessarily to me, but to the patient." OS9

75% of the clinicians supported Flexifoot measuring compliance to clinical advice.

"It would be really useful in terms of keeping a diary of what your patients are doing, especially with the osteoarthritis patients." PT5

"It is helpful if you have any doubts as to whether the patients are being compliant, if they are doing too much or too little activity." OS10

For peer review only

Clinicians can use the feedback as a screening tool, and to help determine the next steps for patient management.

"You could use that as some kind of screening test…do they really need to have a knee replacement yet?" OS1

"In conjunction with physiotherapists…so if you were trying to get them to do a particular rehabilitation programme. Monitor what they're doing, that might be very useful." OS6.

However, GPs felt that Flexifoot was a tool to be used more by patients, rather than by GPs for planning patient care: *"an intervention that's positive for the patient, as opposed to this being an investigation" GP2,* and that feedback would be better interpreted by clinicians with greater musculoskeletal knowledge.

"I can't see any acute use for it that's going to change the patient's management right this second now." GP2

"I'm not sure whether this would change my management for the conditions." GP5

Moreover, various participants in all clinician groups agreed that Flexifoot may be more effective not as a stand-alone device, but to enhance current methods for diagnosis and prognosis, as an *"adjunctive thing to what I already have" P3*.

Specific Measurements

Due to the vast array of parameters that can be analysed after Flexifoot use, it was important to determine the most clinically appropriate specific measurements. Participants were prompted by suggestions of symmetry, stride length, centre of pressure, pressure profiles or ground reaction force. The ideal specific measurements differed slightly between clinician groups due to varying levels of knowledge, but there was a convergence of agreed parameters to be measured by Flexifoot in all clinician groups (Figure 2). GPs provided fewer preferable specific measurements due to gaps in their specialist knowledge regarding musculoskeletal disorders.

The clinicians who supported the monitoring of activity levels (type and number of steps) demonstrated its importance for non-OA conditions too.

"It would give you an idea of their daily routine and exercise levels and things, particularly if they've got other conditions such as diabetes or cardiovascular disease which can be improved by exercise." GP3

Two clinicians exhibited a cautious view regarding tracking patients' activity outside of clinical environments:

"You've got to be careful with monitoring… you don't want engender this sort of 'we're watching you' big brother idea." PT3

"I do think that there could be some, a little degree of patients being suspicious of you checking up on them and they may question the clinician – why can't you trust what you're telling them, why do they need to see what I'm doing?" GP2.

Duration of Wear

The duration of Flexifoot wear suggested by clinicians varied from single usage at clinic appointments, up to long-term periods of over one year, implying a range of uses. The nature of the tool being able to offer real-time advice allows for its acute use in the clinic. The prolonged use of Flexifoot may be appropriate for patients with chronic diseases, or post-operatively in those undergoing surgical interventions. The disparity between preferred lengths depended on the rationale for patient use. 7 clinicians revealed that the duration of Flexifoot wear would be dependent on what outcomes were to be achieved (PT1, PT6, PT8, P2, OS1, OS6), type of injury (PT10), and patient age and compliance (GP5).

For peer review only

"If we were using it as a 'how do they move', we want a picture of their footprint, 5 minutes as they walk along the corridor." PT1

"During periods of activity, if they're experiencing pain or there is a particular challenging part of their day…short bursts of time which are critical to look at, so definitely not all day." OS3

However, long-term monitoring was preferred by most clinicians, where data collection would span over days, weeks, months and years, or *"for as long as it took to establish a meaningful change" PT3.*

"For 24hours or a few days…you'd want the results of this to reflect what they normally do." GP3

A short period of time using the device but at longer intervals between uses was also suggested:

"Snapshots at certain periods, much like we do at follow-up, at 6 weeks, 3 months, and a year." OS4

To maximise the personalised use of Flexifoot, clinicians and patients must collaborate to choose an appropriate duration of wear for each particular case.

Data Presentation

Data Access

50% of the clinicians would prefer to access the data by logging into a system, and some expressed that it should be integrated amongst patients' current notes for information crossover.

"If there was a way of tying in the results so that when you log in and click on the patient, it came up on their results. That would be the most ideal way." GP3

5 clinicians preferred the data via email, although 3 clinicians stated that maintaining patient confidentiality was paramount.

"*Emailed is easier, but then log-in would be better because safety aspects and confidentiality." P2*

Visual Presentation

The data should be concise so that clinicians can quickly interpret data. The clinicians expressed differing opinions after they were prompted with suggestions of graphs, tables and pictures. Therefore, numerous display options should be available to accommodate for all preferences.

"We need to have a summary that is brief, because you don't want to look through tons and tons of data. Then, if you wanted to look into more detail, then there should be the option." GP4

The data should be easily comprehended by patients too. A visually appealing approach with an additional colour-system scheme can enhance patient understanding.

"Patients want to know as well, they want a variation that's patient friendly…an easier format for patients to understand." OS6.

The addition of normal reference ranges alongside objective measures allows for the comparison of patient parameters versus reference data; this will enhance user-experiences and allow for goals to be set.

"Patient specific – it would be graphs. It would be nice to have a normal distribution and see where they fit inside the normal distribution." OS9

Frequency

The clinicians want to receive and access data at appropriate times, such as immediately before or during appointments. The majority (28/30) of clinicians were unlikely to monitor patients outside of the clinic due to time-restraints.

"*I'd access the data just prior to the patient coming in or during that appointment. We would only really have time to monitor or review the data when the patient is actually here." PT5*

"Real-time is helpful for the patient in a therapy session. If they had information once a week on how they're doing. Alerts are ideal, but there is no time to check it." PT10

For peer review only

The generation of data should occur in a timely fashion, *"correlated with the patient's clinic appointment" OS9.* The frequency of data received should depend on personal clinician preferences and the purpose of Flexifoot use.

"There should be an option for the data to be accessible when you want it and you choose." GP5

Barriers to Use

Clinicians highlighted obstacles to implementing Flexifoot (Table 2).

Table 2: The barriers to the use of implementing Flexifoot in clinical practice identified by 30 clinicians

**Barriers to the Use of Flexifoot**
• Time
• Cost and availability
• Influence on practice
• Training/education required
• Patient compliance
• Hygiene control

50% of the clinicians illustrated that time restraints were the largest concern of Flexifoot use clinically, including time taken for initial patient assessments, device introduction, and data generation.

"For me to use this for one patient, explain how to use it and monitor their activity is probably unlikely and unrealistic given the general practice workload and increasing demands on GPs." GP2

"That is the difficulty with this, it is an additional investigation that we need to spend time assessing." OS11

The second most identified barrier was cost – *"it has to be suitably priced…that an average practice can afford" P1.*

The inability of clinicians and patients to interpret data and the training required was another issue. 5 GPs stressed that Flexifoot was too specialised for their environment.

"Patients might not understand the data…people are not familiar with technologies, but this will be less of a problem in the future." PT9

"If it's mainly biometrics and gait analysis…I don't think that I would see this as being within a GP's remit so much. The biggest barrier for me not using it is, identifying how the information it gives would fall into my remit, and how it would influence my practice". GP3

Poor patient compliance also hinders Flexifoot's prospective use.

"Patients are so unreliable and I wouldn't be confident that they would remember to transfer it to another pair of shoes or if they take their shoes off and we'd not be tracking anything." OS3

6 clinicians identified hygiene and infection risks if the device was used for long periods, or between different patients. Flexifoot use between multiple patients could be more economically practical however.

"I suppose you've got questions of hygiene…you'd need a material you can wipe and maybe some way of cleaning them really well, so they would be to an infection control standard. If you can use them more than once, it would be cost effective." P3

Future Development

Following the responses to the set questions, clinicians offered suggestions on how to optimise Flexifoot for successful clinical uptake.

For peer review only

Parameters that would be ideally measured using Flexifoot (Figure 2) could be adapted to measure more factors and expand patient target audiences. Clinicians suggested Flexifoot use in Parkinson's disease, peripheral neuropathy in diabetes, chronic pain conditions, obesity, hemiplegia and tendinopathies.
"Pressure profiles are good, but it'd be really great to measure shear and temperatures in the foot…it would be better and more useful for people with plantar foot pain. Diabetics – it would be great." OS11

Design changes were also proposed, *"maybe this ribbon (ankle strap) could be a bit smaller because it might irritate someone on the side and it might artificially affect their gait" OS10.*

DISCUSSION

A technology must be user-friendly to optimise its efficacy and sustainability[16,17]. We conducted structured interviews with clinicians to guide the development of our novel technology, Flexifoot, towards clinical uptake. The clinicians expressed numerous advantages of adopting Flexifoot into healthcare settings and barriers, indicating strategies for future improvement. The main advantage recognised by clinicians was the ability of Flexifoot to generate quantitative data, that can be used for monitoring and feedback in various clinical contexts.

Clinicians implied that Flexifoot would not replace current clinical tools, but instead complement them. The ambulatory quantitative data can support existing OA diagnostic and management tools, and help to improve the reliability of clinical decisions. The ambulatory monitoring of disease progression, alongside patients' responses to treatment, has been considered useful[18]. Other rehabilitation technologies that motivate and offer objective feedback have been associated with long-term benefits and good physical fitness levels[19]. Despite this, some lower limb wearable technologies for rehabilitation have been described to have limited efficacy for the improvement of activity levels, but mainly due to poor research methodologies used in the past[20]. However, there is a still a demand for self-management of rehabilitation with feedback using shoe insole pressure sensors[21]. The contradictory results in the literature regarding wearable technologies calls for a study that explores the users' perspectives to maximise acceptance.

Moreover, objective feedback can enable a more efficient exchange and handover of patient information between clinicians[22]**.** A clinician may use the tool as a screening approach in adjunction to current methods, for more reliable results and subsequently refer the patient onto a specialist[14]. Also, the clinicians recognised that the data can reinforce their dialogue with patients, making patients more aware of their problem. Awareness and feedback was seen as a way to enhance patient self-management. Tracking activity levels and feedback engages patient involvement in their own care, and is useful for other non-musculoskeletal chronic conditions too[23,24].

Clinicians, in our study, indicated how Flexifoot could be a feedback tool for patients. The data being available to patients allows for greater independence in self-monitoring and feedback of their own diseases in familiar environments outside of clinics[18]. Home-based training and monitoring devices showed higher patient satisfaction compared to similar care within clinics[25]. Self-management can also reduce economic burdens as the technology can educate OA patients, improve outcomes and reduce hospital visits[18,26].

The uses and specific measurements suggested by clinicians was greatly dependent on the type of clinician and specific patient cohorts, which is also the case for other musculoskeletal interventions[27]. The GPs felt that they had insufficient gait analysis knowledge, and that the device was presently too specialist for general practice. GP environments may be inappropriate since it comprises of too broad a range of patients. Instead, they indicated that Flexifoot would be better suited for clinicians that follow up patients more regularly. This was reiterated by physiotherapists, orthopaedic surgeons and podiatrists, who are better equipped in musculoskeletal fields, and expressed positivity for Flexifoot's clinical uptake.

For peer review only

All clinicians indicated that the system and data output should be easy to use and interpret. Ease of use of wearable sensors for clinicians was reported in the past to facilitate their adoption into clinical settings[28]. The prospect of a log-in software system for Flexifoot data was more popular than receiving results via email. The interface should be integrated alongside current patient records for information crossover and a choice of data presentation styles should be available. The material should be presented alongside normal reference ranges, for comparisons and targets to be made. Data accessibility and presentation should be understood by patients too – this is key for patient acceptance and accessibility[29]. Clinicians' views obtained in another study recognised that shorter, simpler and more concise data as educational material is preferable for patients, but that detailed data should be fully available too[30]. This agrees with our findings: participants expressed the possibility of having access to more detailed data if needed, whilst avoiding scanning excessive data beyond their understanding. Full data measurements could be stored however for more skilled users in research settings[31].

Furthermore, shortcomings of Flexifoot were recognised by the clinicians which may explain why the practical application of similar insole monitoring devices have not been successful in the past[32].

The clinicians' continuous workloads means that using real-time data from Flexifoot is only feasible prior to or during appointments in the presence of patients. The real-time data and automatic alerts may be more useful for patient users for receiving feedback, which continues to motivate them[13]. Clinicians are reluctant for the introduction of new tools because they can disrupt time-pressured practice schedules, and the time required to train them to use Flexifoot must also be considered[33]. In the past, numerous medical wearable technologies for a range of users have failed to meet the criteria of being simple and powerful in terms of data output and energy consumption[32]. However, although clinicians may perceive new tools as a hindrance, a study showed that adults suffering from osteoarthritis felt that more novel approaches could be implemented for the management of their condition[34,35]. The clinical efficacy of Flexifoot must be therefore fully established, such as through patient usability testing, before clinicians can adopt it as a method worthy of appointment time.

High costs also limit new technology implementation within health services. The current expense to manufacture one Flexifoot device is low, but one tool per patient may be economically impractical. The recycling and reuse of devices between patients may reduce costs, but increases hygiene and infection risks, expressed by 6 clinicians. Introducing hygienic procedures prior to and after Flexifoot use could enhance its reusability and cost-effectiveness. Thus, the cost-effectiveness, with reference to current treatment guidelines, should be further investigated. Patient compliance is also an issue since long-term wearable devices require adequate patient acceptability. However, from a similar study we conducted that explored patients' views, all participants were keen for the uptake of wearable technologies[13]. Moreover, effective clinician-patient communication can determine patient cohorts supportive of self-management, and those more likely to adhere to using Flexifoot[13,14,36]. Clinicians can promote the relevance of sensor technologies for patients' care, and hence boost compliance[12,37]. The identified issues surrounding Flexifoot are apparent in other wearable technologies too[27]. The replication of problems between devices implies a necessity for new approaches in encouraging patients' compliance and appeal for novel strategies. The findings that emerged from this study can be translated to other similar technologies to promote their clinical uptake and foster new developments.

The study limitations involved the clinicians' varied levels of experience and familiarity of instrumented insoles, and that 27/30 interviewees were based in the London area. The clinicians had not used Flexifoot, and telephone interviews could not view the device, but detailed descriptions and commercially similar devices that could be found online were provided prior to interviews. Future studies would involve clinicians' use of Flexifoot beforehand. Moreover, clinicians who were well-informed around the subject were perhaps biased to initially participate and express positivity. However, the interviews were confidential and honest feedback was encouraged.

In conclusion, the clinicians considered Flexifoot to be a useful tool that could be used in adjunction to current approaches, in a long-term, follow-up setting to support and improve patient care. The clinicians' preferences exhibited numerous ways in which Flexifoot can be useful for patients with OA or other

For peer review only

conditions. The measured parameters should be selected according to patient-specific cases, and delivered in a concise manner through a secure interface. A choice of data outputs should be offered to cater for all users. The challenges of time, cost, infection control should be addressed, alongside the clinical efficacy and cost-effectiveness for the clinical adoption of Flexifoot and similar technologies.

Author Contributions: DL, EP, and AHM conceived and designed the study. DL carried out the interviews; DL and EP analysed and interpreted the data. DL and EP drafted the manuscript. All authors read, edited, and approved the final version of the manuscript. All authors had full access to all of the data in the study and can take responsibility for the integrity of the data and the accuracy of the data analysis.

Acknowledgement: We would like to thank MSc student Mary Goodwin for her contribution with data collection.

Funding: This research received no specific grant from any funding agency in the public, commercial or not-for-profit sectors

Competing interests: The authors declare that there are no conflicts of interest.

All authors have completed the ICMJE uniform disclosure form at www.icmje.org/coi_disclosure.pdf (available on request from the corresponding author) and declare: no support from any organisation for the submitted work; no financial relationships with any organisations that might have an interest in the submitted work in the previous three years; no other relationships or activities that could appear to have influenced the submitted work.

Ethical approval: The study was reviewed and approved by Imperial College London Ethics Research Committee. All participants gave written informed consent.

Data sharing: No additional data available.

References

1 Bennell KL, Hunter DJ, Hinman RS. Management of osteoarthritis of the knee. *Bmj* 2012;**345**:e4934–e4934. doi:10.1136/bmj.e4934

2 Simon D, Mascarenhas R, Saltzman BM, *et al.* The Relationship between Anterior Cruciate Ligament Injury and Osteoarthritis of the Knee. *Adv Orthop* 2015;**2015**. doi:10.1155/2015/928301

3 Muthuri SG, McWilliams DF, Doherty M, *et al.* History of knee injuries and knee osteoarthritis: A meta-analysis of observational studies. *Osteoarthr Cartil* 2011;**19**:1286–93. doi:10.1016/j.joca.2011.07.015

4 Driban JB, Eaton CB, Lo GH, *et al.* Association of Knee Injuries With Accelerated Knee Osteoarthritis Progression: Data From the Osteoarthritis Initiative. *Arthritis Care Res* 2014;**66**:1673–9. doi:10.1002/acr.22359

5 Jones CA, Voaklander DC, Johnston DW, *et al.* Health related quality of life outcomes after total hip and knee arthroplasties in a community based population. *J Rheumatol* 2000;**27**:1745–52.http://www.ncbi.nlm.nih.gov/pubmed/10914862 (accessed 29 Mar 2018).

6 Wylde V, Hewlett S, Learmonth ID, *et al.* Persistent pain after joint replacement: Prevalence, sensory qualities, and postoperative determinants. *Pain* 2011;**152**:566–72. doi:10.1016/j.pain.2010.11.023

7 Singh JA, O'Byrne M, Harmsen S, *et al.* Predictors of moderate-severe functional limitation after primary Total Knee Arthroplasty (TKA): 4701 TKAs at 2-years and 2935 TKAs at 5-years. *Osteoarthr Cartil* 2010;**18**:515–21. doi:10.1016/j.joca.2009.12.001

8 Dowsey MM, Nikpour M, Dieppe P, *et al.* Associations between pre-operative radiographic changes and outcomes after total knee joint replacement for osteoarthritis. *Osteoarthr Cartil* 2012;**20**:1095–102. doi:10.1016/j.joca.2012.05.015

9 Elbaz A, Mor A, Segal G, *et al.* Novel classification of knee osteoarthritis severity based on spatiotemporal gait analysis. *Osteoarthr Cartil* 2014;**22**:457–63. doi:10.1016/j.joca.2013.12.015

10 Harris KK, Dawson J, Jones LD, *et al.* Extending the use of PROMs in the NHS—using the Oxford Knee Score in patients undergoing non-operative management for knee osteoarthritis: a validation study. *BMJ Open* 2013;**3**:e003365. doi:10.1136/bmjopen-2013-003365

11 Roos EM, Roos HP, Lohmander LS, *et al.* Knee Injury and Osteoarthritis Outcome Score (KOOS)—

For peer review only

Development of a Self-Administered Outcome Measure. *J Orthop Sport Phys Ther* 1998;**28**:88–96. doi:10.2519/jospt.1998.28.2.88

12 Bergmann JHM, McGregor AH. Body-worn sensor design: What do patients and clinicians want? *Ann Biomed Eng* 2011;**39**:2299–312. doi:10.1007/s10439-011-0339-9

13 Papi E, Belsi A, McGregor AH. A knee monitoring device and the preferences of patients living with osteoarthritis: a qualitative study. *BMJ Open* 2015;**5**:e007980. doi:10.1136/bmjopen-2015-007980

14 Papi E, Murtagh GM, McGregor AH. Wearable technologies in osteoarthritis: a qualitative study of clinicians' preferences. *BMJ Open* 2016;**6**:e009544. doi:10.1136/bmjopen-2015-009544

15 Ritchie J, Lewis J. QUALITATIVE RESEARCH PRACTICE A Guide for Social Science Students and Researchers. https://mthoyibi.files.wordpress.com/2011/10/qualitative-research-practice_a-guide-for-social-science-students-and-researchers_jane-ritchie-and-jane-lewis-eds_20031.pdf (accessed 13 Dec 2017).

16 Lewis JE, Neider MB. Designing Wearable Technology for an Aging Population. *Ergon Des* 2017;**25**:4–10. doi:10.1177/1064804616645488

17 Lee J, Kim D, Ryoo H-Y, *et al.* Sustainable Wearables: Wearable Technology for Enhancing the Quality of Human Life. *Sustainability* 2016;**8**:466. doi:10.3390/su8050466

18 Majumder S, Mondal T, Deen M. Wearable Sensors for Remote Health Monitoring. *Sensors* 2017;**17**:130. doi:10.3390/s17010130

19 Aamot I-L, Karlsen T, Dalen H, *et al.* Long-term Exercise Adherence After High-intensity Interval Training in Cardiac Rehabilitation: A Randomized Study. *Physiother Res Int* 2016;**21**:54–64. doi:10.1002/pri.1619

20 Powell L, Parker J, Martyn St-James M, *et al.* The Effectiveness of Lower-Limb Wearable Technology for Improving Activity and Participation in Adult Stroke Survivors: A Systematic Review. *J Med Internet Res* 2016;**18**:e259. doi:10.2196/jmir.5891

21 Munoz-Organero M, Parker J, Powell L, *et al.* Assessing Walking Strategies Using Insole Pressure Sensors for Stroke Survivors. *Sensors (Basel)* 2016;**16**. doi:10.3390/s16101631

22 Popovici I, Morita PP, Doran D, *et al.* Technological aspects of hospital communication challenges: An observational study. *Int J Qual Heal Care* 2015;**27**:183–8. doi:10.1093/intqhc/mzv016

23 Chiauzzi E, Rodarte C, DasMahapatra P. Patient-centered activity monitoring in the self-management of chronic health conditions. *BMC Med* 2015;**13**:1–6. doi:10.1186/s12916-015-0319-2

24 Thornton JS, Frémont P, Khan K, *et al.* Physical activity prescription: a critical opportunity to address a modifiable risk factor for the prevention and management of chronic disease: a position statement by the Canadian Academy of Sport and Exercise Medicine. *Br J Sports Med* 2016;**50**:1109–14. doi:10.1136/bjsports-2016-096291

25 Kraal JJ, Elske Van Den Akker-Van Marle M, Abu-Hanna A, *et al.* Clinical and cost-effectiveness of home-based cardiac rehabilitation compared to conventional, centre-based cardiac rehabilitation: Results of the FIT@Home study. *Eur J Prev Cardiol* 2017;**24**:1260–73. doi:10.1177/2047487317710803

26 Bodenheimer T, Lorig K. Patient Self-management of Chronic Disease in Primary Care. 2014;**288**:2469–75. doi:10.1001/jama.288.19.2469

27 de Almeida PHTQ, MacDermid JC, Pontes TB, *et al.* Orthotic use for CMC osteoarthritis: Variations among different health professionals in Brazil. *J Hand Ther* 2016;**29**:440–50. doi:10.1016/j.jht.2016.07.007

28 Grimm B, Bolink S. Evaluating physical function and activity in the elderly patient using wearable motion sensors. *EFORT Open Rev* 2016;**1**:112–20. doi:10.1302/2058-5241.1.160022

29 Puri A, Kim B, Nguyen O, *et al.* User Acceptance of Wrist-Worn Activity Trackers Among Community-Dwelling Older Adults: Mixed Method Study. *JMIR mHealth uHealth* 2017;**5**:e173. doi:10.2196/mhealth.8211

30 Grudniewicz A, Bhattacharyya O, McKibbon KA, *et al.* User-centered design and printed educational materials: A focus group study of primary care physician preferences. *J Contin Educ Health Prof* 2016;**36**:249–55. doi:10.1097/CEH.0000000000000112

31 Iqbal MH, Aydin A, Brunckhorst O, *et al.* A review of wearable technology in medicine. *J R Soc Med* 2016;**109**:372–80. doi:10.1177/0141076816663560

32 Haghi M, Thurow K, Stoll R. Wearable Devices in Medical Internet of Things: Scientific Research and

For peer review only

Commercially Available Devices. *Healthc Inform Res* 2017;**23**:4–15. doi:10.4258/hir.2017.23.1.4

33 Yarbrough a. K, Smith TB. A new take on TAM. *Med Care Res Rev* 2007;**64**:650–72. doi:10.1177/1077558707305942

34 Mackay C, Hawker GA, Jaglal SB. Qualitative study exploring the factors influencing physical therapy management of early knee osteoarthritis in Canada. *BMJ Open* 2018;**8**:23457. doi:10.1136/bmjopen-2018-023457

35 MacKay C, Sale J, Badley EM, *et al.* Qualitative Study Exploring the Meaning of Knee Symptoms to Adults Ages 35-65 Years. *Arthritis Care Res (Hoboken)* 2016;**68**:341–7. doi:10.1002/acr.22664

36 Loos JR, Davidson EJ. Wearable health monitors and physician-patient communication: The physician's perspective. *Proc Annu Hawaii Int Conf Syst Sci* 2016;**2016–March**:3389–98. doi:10.1109/HICSS.2016.422

37 Teh RC-A, Mahajan N, Visvanathan R, *et al.* Clinical effectiveness of and attitudes and beliefs of health professionals towards the use of health technology in falls prevention among older adults. *Int J Evid Based Healthc* 2015;**13**:213–23. doi:10.1097/XEB.0000000000000029

Supplementary files: Semi-structured interview questions for clinicians

Figure legends:

Figure 1: a) Layout of pressure sensors on the insoles with connectors for the circuit boards. b) Insoles covered with neoprene with circuit boards for data transmission attached and ready to be inserted into shoes.

Figure 2: Clinician preferences for the specific measurements that they would like Flexifoot to obtain.

For peer review only

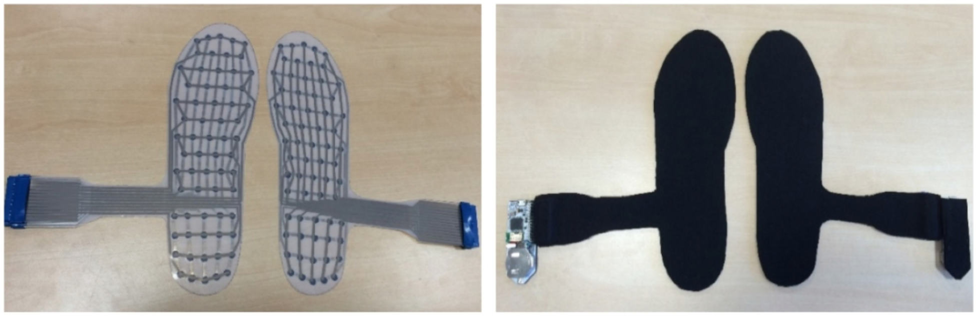

a b

Figure 1: a) Layout of pressure sensors on the insoles with connectors for the circuit boards. b) Insoles covered with neoprene with circuit boards for data transmission attached and ready to be inserted into shoes.

157x62mm (200 x 200 DPI)

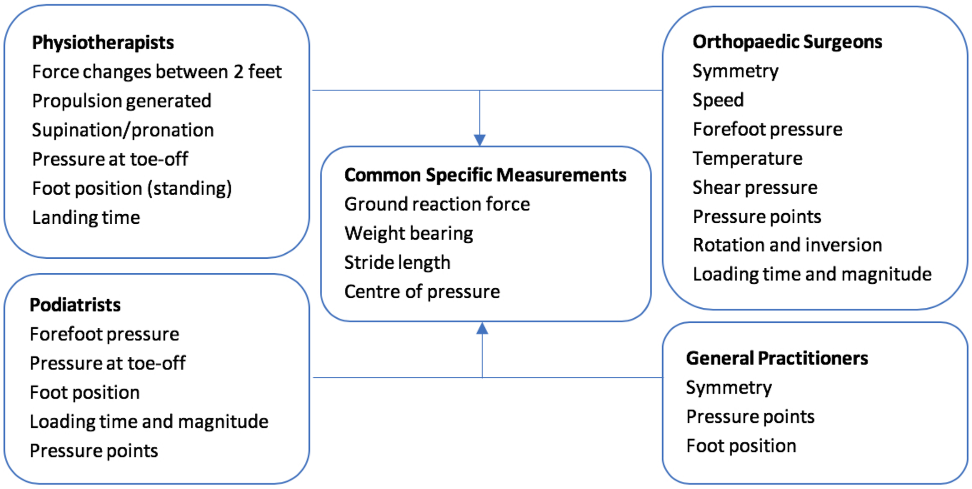

Figure 2: Clinician preferences for the specific measurements that they would like Flexifoot to obtain.

152x74mm (200 x 200 DPI)

Semi-structured interview questions for clinicians

Contextual Background

- What is your age and nationality (you can prefer not to say)?
- What is/can you describe your professional role?
- How long have you been in this role?
- What types of patients do you come into contact with?
    A. Is there a particular type/category e.g OA/ACL?
    B. Does it vary?
    C. How many patients do you see per day?
    D. Is there a particular age range of patients you work with?

Wearable Technologies

- What do you know about wearable technologies?
- Do you currently use any wearable technologies in your work?

Clinician Preferences for the Flexifoot Device (With Reference to OA Surgery and ACL Injuries)

- Would you find Flexifoot useful for your OA and/or ACL injury patients?
- How would such a device help you in your own work? How would you use it?
- What would you specifically like to measure using the Flexifoot device?
- Would you like information about e.g.
    A. the patient's gait, such as symmetry/stride length?
    B. centre of pressure and pressure profiles for subsections of the foot?
    C. ground reaction force?
- Is there any parameters that should be tailored for OA surgery or for ACL injuries patients? (Different measurements used in accordance to the patients?)
- In addition to specific parameters, would you find it useful to measure the activity level of your patients? (e.g. time when active or not)
- How do you feel about monitoring compliance to exercise programmes?
- How often and for how long would you want the patient to wear the device?
- How would you like the data presented to you? E.g. in graphs, summary tables, performance profiles over a certain period of time?
- Would you find it useful to have a brief summary of the patient's progress with the option to look in more detail at certain aspects of the data?
- When would you like the data to be available to you? E.g. every day the patient uses Flexifoot?
- How would you ideally access the data? Would you like it emailed to you or would you prefer to have a website where you can log in to access it?
- Would you find an automatic alert system useful that told you when the data was available from your patient?
- Would you like an alert that will flag up if the metrics you identified fall below a certain threshold? (e.g. the patient is not doing exercise at all, so you need to send them a reminder?)
- Would you find Flexifoot to be more useful for patients with ACL injury or for pre-/post-OA surgery?
- What other information would you use to complement the use of Flexifoot in your clinical practice? Any other parameters that you think will be valuable that cannot be measured with Flexifoot?
- Can you suggest any reasons that may prevent you from using this device should it became available for use in clinical practice?

Closure

- Do you have any other comments about the Flexifoot device?

For peer review only

Standards for Reporting Qualitative Research (SRQR)*

http://www.equator-network.org/reporting-guidelines/srqr/

Title and abstract

	Page/line no(s).
**Title** - Concise description of the nature and topic of the study Identifying the study as qualitative or indicating the approach (e.g., ethnography, grounded theory) or data collection methods (e.g., interview, focus group) is recommended	Page 1: Lines 1-2
**Abstract** - Summary of key elements of the study using the abstract format of the intended publication; typically includes background, purpose, methods, results, and conclusions	Page 1: Lines 25-50

Introduction

**Problem formulation** - Description and significance of the problem/phenomenon studied; review of relevant theory and empirical work; problem statement	Page 2: Lines 15-51
**Purpose or research question** - Purpose of the study and specific objectives or questions	Page 2: Lines 49-54<br>Page 3: Lines 1-6

Methods

**Qualitative approach and research paradigm** - Qualitative approach (e.g., ethnography, grounded theory, case study, phenomenology, narrative research) and guiding theory if appropriate; identifying the research paradigm (e.g., postpositivist, constructivist/ interpretivist) is also recommended; rationale**	Page 3: Line 10-19,<br>Page 3: Lines 5-24-43
**Researcher characteristics and reflexivity** - Researchers' characteristics that may influence the research, including personal attributes, qualifications/experience, relationship with participants, assumptions, and/or presuppositions; potential or actual interaction between researchers' characteristics and the research questions, approach, methods, results, and/or transferability	Page 3: Lines 25-28<br>Page 10: Lines 5-6
**Context** - Setting/site and salient contextual factors; rationale**	Page 3: Lines 11-12, 16-22, 40-43
**Sampling strategy** - How and why research participants, documents, or events were selected; criteria for deciding when no further sampling was necessary (e.g., sampling saturation); rationale**	Page 2: Lines 53-54<br>Page 3: Lines 1-4, 40-43
**Ethical issues pertaining to human subjects** - Documentation of approval by an appropriate ethics review board and participant consent, or explanation for lack thereof; other confidentiality and data security issues	Page 3: Lines 11-13, 40-43
**Data collection methods** - Types of data collected; details of data collection procedures including (as appropriate) start and stop dates of data collection and analysis, iterative process, triangulation of sources/methods, and modification of procedures in response to evolving study findings; rationale**	Page 3: Line 11, 24-37

For peer review only

**Data collection instruments and technologies** - Description of instruments (e.g., interview guides, questionnaires) and devices (e.g., audio recorders) used for data collection; if/how the instrument(s) changed over the course of the study	Page 3: Lines 11, 24-37
**Units of study** - Number and relevant characteristics of participants, documents, or events included in the study; level of participation (could be reported in results)	Page 3: Lines 15-33, 46-48
**Data processing** - Methods for processing data prior to and during analysis, including transcription, data entry, data management and security, verification of data integrity, data coding, and anonymization/de-identification of excerpts	Page 3: Line 11, 24-37
**Data analysis** - Process by which inferences, themes, etc., were identified and developed, including the researchers involved in data analysis; usually references a specific paradigm or approach; rationale**	Page 3: Lines 24-37, 46-51<br>Page 4: 1-2
**Techniques to enhance trustworthiness** - Techniques to enhance trustworthiness and credibility of data analysis (e.g., member checking, audit trail, triangulation); rationale**	Page 3: Lines 34-37

Results/findings

**Synthesis and interpretation** - Main findings (e.g., interpretations, inferences, and themes); might include development of a theory or model, or integration with prior research or theory	Page 3: Lines 46-51<br>Page 4: Lines 1-3
**Links to empirical data** - Evidence (e.g., quotes, field notes, text excerpts, photographs) to substantiate analytic findings	Page 4: Lines 6-42<br>Pages 5-7<br>Page 8: Lines 1-10

Discussion

**Integration with prior work, implications, transferability, and contribution(s) to the field -** Short summary of main findings; explanation of how findings and conclusions connect to, support, elaborate on, or challenge conclusions of earlier scholarship; discussion of scope of application/generalizability; identification of unique contribution(s) to scholarship in a discipline or field	Page 8: Lines 13-52<br>Page 9: Lines 1-50
**Limitations** - Trustworthiness and limitations of findings	Page 9: Lines 35-41

Other

**Conflicts of interest** - Potential sources of influence or perceived influence on study conduct and conclusions; how these were managed	Page 10: Lines 7-12
**Funding** - Sources of funding and other support; role of funders in data collection, interpretation, and reporting	Page 10: Lines 5-6

For peer review only - http://bmjopen.bmj.com/site/about/guidelines.xhtml

*The authors created the SRQR by searching the literature to identify guidelines, reporting standards, and critical appraisal criteria for qualitative research; reviewing the reference lists of retrieved sources; and contacting experts to gain feedback. The SRQR aims to improve the transparency of all aspects of qualitative research by providing clear standards for reporting qualitative research.

**The rationale should briefly discuss the justification for choosing that theory, approach, method, or technique rather than other options available, the assumptions and limitations implicit in those choices, and how those choices influence study conclusions and transferability. As appropriate, the rationale for several items might be discussed together.

Reference:

O'Brien BC, Harris IB, Beckman TJ, Reed DA, Cook DA. **Standards for reporting qualitative research: a synthesis of recommendations.** *Academic Medicine*, Vol. 89, No. 9 / Sept 2014 DOI: 10.1097/ACM.0000000000000388

For peer review only

BMJ Open

Exploring the clinical context of adopting an instrumented insole: a qualitative study of clinicians' preferences in England

Journal:	*BMJ Open*
Manuscript ID	bmjopen-2018-023656.R3
Article Type:	Research
Date Submitted by the Author:	06-Mar-2019
Complete List of Authors:	Lin, Denise; Imperial College London Department of Surgery and Cancer<br>Papi, Enrica; Imperial College London, Department of Surgery and Cancer<br>McGregor, Alison; Imperial College London, Dept of Surgery and Cancer
**Primary Subject Heading**:	Qualitative research
Secondary Subject Heading:	Rehabilitation medicine
Keywords:	wearable technologies, Osteoarthritis, Self-managment, Pressure sensors, Users' preferences

SCHOLARONE™
Manuscripts

Exploring the clinical context of adopting an instrumented insole: a qualitative study of clinicians' preferences in England

Denise Lin[a], Enrica Papi[a,b], Alison McGregor[a]

[a]Department of Surgery and Cancer, Imperial College London, London, UK
[b] Department of Bioengineering, Imperial College London, London, UK

Corresponding author:
Dr Enrica Papi
Department of Surgery and Cancer,
Imperial College London,
Room 7L16, Floor 7, Laboratory Block,
Charing Cross Hospital,
London, W6 8RF, UK
Email: e.papi@imperial.ac.uk
Phone: 020 3313 8833

Denise Lin: email: denise.lin14@imperial.ac.uk

Alison H McGregor: email: a.mcgregor@imperial.ac.uk

Keywords: wearable technologies, osteoarthritis, self-management, users preferences, pressure sensors
Words Count: 4186

ABSTRACT

Objectives: This study explores clinicians' views of the clinical uptake of a smart pressure-sensing insole, named Flexifoot, to enhance the care and management of patients with osteoarthritis (OA). Clinicians are key users of wearable technologies, and can provide appropriate feedback for a specific device for successful clinical implementation.

Design: Qualitative study with in-depth, semi-structured interviews, analysed using inductive analysis to generate key themes.

Setting: Conducted in a University setting.

Participants: 30 clinicians were interviewed (11 physiotherapists, 11 orthopaedic surgeons, 5 general practitioners, 3 podiatrists).

Results: All clinicians regarded Flexifoot to be useful for the care and management of patients in adjunction to current methods. Responses revealed four main themes: use, data presentation, barriers to use, and future development. Flexifoot data was recognised as capable of enhancing information exchange between clinicians and patients, and also between clinicians themselves. Participants supported the use of feedback for rehabilitation, screening and evaluation of treatment progress/success purposes. Flexifoot use by patients was encouraged as a self-management tool that may motivate them by setting attainment goals. The data interface should be secure, concise and visually appealing. The measured parameters of Flexifoot, its duration of wear and frequency of data output would all depend on the rationale for its use. The clinicians and patients must collaborate to optimise the use of Flexifoot for long-term monitoring of disease for patient care in clinical practice. Many identified potential other uses for Flexifoot.

Conclusions: Clinicians thought that Flexifoot may complement and improve current methods of long-term patient management for OA or other conditions in clinical settings. Flexifoot was recognised to be useful for objective measures and should be tailored carefully for each person and condition to maximise compliance. Adopting the device, and other similar technologies, requires reducing the main barriers to use (time, cost, patient compliance) before its successful implementation.

Strengths and limitations of this study

- This was the first qualitative study to specifically explore clinicians' views on implementing an in-house smart, flexible, pressure-sensing insole tool into clinical practice for patients with osteoarthritis
- The views of clinicians were fully explored with in-depth interviews, and analysed with inductive thematic analysis, giving rise to detailed suggestions to optimise the device's role alongside current strategies in patient care
- Clinicians were unable to use the device themselves prior to the interviews and responses were based upon one single demonstration and explanation of the tool
- Clinicians had a varied level of experience and familiarity of wearable technologies between them, influencing their perspectives

INTRODUCTION

Osteoarthritis (OA) is one of the most common long-term musculoskeletal diseases, and cause of pain and functional disability[1]. Individuals who have sustained a knee injury, such as anterior cruciate ligament (ACL) injuries, are 3-6 times more likely to develop knee OA[2–4]. In these patients, diagnosis occurs approximately 10 years prior to those without a previous injury[3,4], calling for long-term management of such conditions. Current clinical guidelines recommend physical activity to delay surgical intervention that is known to have a limited lifespan and in many instances poor reported patient outcomes[5–8], despite the belief that joint replacement is one of the most successful surgical procedures. Conversely, poor patient compliance limits long-term exercise benefits for OA, and many disregard the benefits of exercise.

Pain and gait changes are reasons why OA patients primarily visit clinicians. Gait analysis helps to establish OA diagnoses, severity and biomechanics underpinning musculoskeletal disorders[9]. In clinical settings, however, patient gait is observed by the clinician's eye, and self-reported questionnaires, such as the *36-Health Survey (SF-36)*, *Western Ontario and McMaster Universities Osteoarthritis Index (WOMAC)*, *Oxford Knee Score (OKN)* and *Knee Osteoarthritis Outcome Scores (KOOS)*, can assess OA severity[9–11]. Gait monitoring through clinician observation and patient questionnaires are prone to subjective responses, and therefore, are inadequate methods to quantify symptoms.

The emergence of wearable technologies can enhance current tools of physiotherapy, rehabilitation and daily monitoring of physical activity. Novel, portable wearable technologies offer a promising approach for use outside of the laboratory, to monitor functional changes in disease progression and activity levels. Nevertheless, the clinical implementation of wearable technologies is seemingly difficult. To enhance translation into clinical context, patient and clinicians' preferences have been explored in the past to determine the views and criteria of users for wearable technologies[12]. OA patients revealed that tracking disease progress was appealing and encouraged exercise[13]. Thirteen clinicians supported wearable technologies for OA patients clinically, but the preferences provided did not have specificity to one device[14].

Within our group, we developed a smart, flexible, pressure-sensing insole, aptly named 'Flexifoot' (Figure 1). Flexifoot generates plantar pressure readings from various foot regions. A high-resolution pressure map can be created from data that feeds back wirelessly to a smartphone app, for the extrapolation of gait spatio-temporal parameters, centre of pressure and pressure distribution. Flexifoot, being portable and low-cost to manufacture (approximately £20), in contrast to laboratory based-force-platforms, allows for continuous data collection over a substantial number of gait cycles, for feedback to patients and clinicians as needed. Daily gait and pressure analysis can enable patients to monitor improvements and disease-related progression, as well as guide clinicians through treatment decisions. Flexifoot is yet to be validated, therefore exploring users' preferences is beneficial for its ongoing development.

The ambiguity of previously obtained clinician preferences lacks the definitive feedback required to improve the design of a specific tool. The lack of specificity can be addressed by probing more into the

details of the clinical implementation of Flexifoot for OA and other disorders. The diagnosis and management of OA involves a multitude of healthcare professional types and it is therefore important to understand how Flexifoot could best address their requirements to inspire design and outcome measures which will facilitate clinical uptake. The aims of this study were to explore the clinicians' preferences for their use of Flexifoot and to identify specific parameters to be measured by the tool to foster improvements, and ultimately enhance OA patient care.

METHODS

Study Design

The study was a qualitative study based on in-depth semi-structured interviews with 30 clinicians. The study was reviewed and approved by Imperial College London Ethics Research Committee. All participants gave written informed consent prior to participation.

Participants

30 clinicians (18 males and 12 females, aged 21-57), including 11 physiotherapists, 11 orthopaedic surgeons, 5 general practitioners (GPs) and 3 podiatrists, were recruited for one-to-one interviews.

Clinicians that had previously or currently worked for the Imperial College Healthcare National Health Service (NHS) Trust were invited via telephone and email invitations to partake in our study. The recruitment began from October 2015 and interviews occurred until September 2017, when data saturation occurred.

At the time of clinician interviewing, the healthcare professionals practiced amongst private and NHS settings within London and Greater London, one in Hereford, one in Cheltenham and one in Liverpool. They had from 4 months up to 28 years of experience within current specialities.

Interviews and Data

The interviews were performed by researchers (DL and MG) in person, except for 2, which were conducted over the telephone due to scheduling constraints. The researchers did not have any personal relationships with the study participants, and the group had prior experience in conducting qualitative studies[13,14]. Face-to-face interviews were audio-recorded and transcribed afterwards.

Prior to each interview, participants' consent was obtained and researchers explained project aims, described Flexifoot and showed a prototype to each clinician (except in telephone interviews). Open-ended questions prompted clinicians to explore perspectives regarding the relevance of Flexifoot in clinical practice. The interview questions (Supplementary File 1) highlighted Flexifoot's clinical influence, specific measurements, data presentation preferences, and gave scope for feedback and improvement.

The interview verbatim transcriptions were analysed using inductive thematic analysis[15], without prior theoretical influences, whereby key findings were analysed and collated into early themes by DL and EP separately. DL and EP then checked each other's data and themes, ensuring consistency and the generation of recurrent key themes.

Patient and Public Involvement

While patients and users were not directly involved in the design of this study, this study arose from previous work where patients highlighted that their views were not considered in the design of novel wearable devices, thereby limiting uptake and translation[13,14]. This study directly focuses on care practitioners' preferences and requirements.

RESULTS

The semi-structured interviews opened with questions to determine healthcare backgrounds of clinicians and the relevance of wearable technologies within their profession. 24 out of 30 clinicians were aware of wearable technologies, and 4 used them for patients.

For peer review only

Inductive analysis[15] of interview responses revealed four main themes, with sub-themes: use (applications, specific measurements, duration of wear), data presentation (data access, visual presentation, frequency of data), barriers to use and future development. The former three themes surfaced from specific interview questions asked. The latter was brought about after clinicians offered feedback as to how Flexifoot could be improved. The themes will be hereinafter described and verbatim quotes are indicated by: PT (physiotherapists), OS (orthopaedic surgeons), P (podiatrists) and GPs (general practitioners), followed by randomly assigned numbers.

Uses

Applications

The main uses of Flexifoot identified by clinicians are shown in Table 1.

Table 1: The main five applications of Flexifoot in clinical practice identified by 30 clinicians

**Main Applications of Flexifoot in Clinical Practice**
• Assessing efficacy of treatment (pre- and post-treatment)
• Monitoring disease progression
• Feedback for patients and other clinicians
• Monitoring activity levels and compliance
• Screening test to support future management

All groups of clinicians recognised Flexifoot as an objective outcome measure tool to monitor various parameters. 23 clinicians expressed that Flexifoot objective measures are useful to assess symptoms, and progress before and after medical intervention.

"It would be useful as an objective outcome measure for change…assess patients at time intervals for pre- and post-surgery." PT7

"This would be very interesting for research or pre- and post-surgery because you'd be able to monitor and look at change…it would definitely be useful as a follow-up guidance to surgical correction." OS11

21 clinicians felt that objective data can reinforce clinical interpretations and enhance information exchange between healthcare professionals. Moreover, real-time objective feedback can help to visually demonstrate the problem and solutions to patients.

"For us feeding back to the surgeons…you can be a bit more accurate about what it is that you're saying." PT11

"It might be useful to demonstrate to the patient what some of their symptoms are. To give them a visual representation of that, I could show them this while they're walking." OS10

"It's important for the patients to visualise what the problem is…as a relatively low-grade, without major intervention, you could do a lot with it to see how to correct problems objectively…It may then allow them to see visually what the issue is, so that if they correct it with the help of someone." GP2

Clinicians recognised Flexifoot as a self-management tool: rehabilitation targets can be set by patients themselves, or by clinicians, and motivate patients.

"For patients to use at home as a rehabilitation tool to set targets or goals they can monitor themselves." PT8

"Anything that can give feedback to the patient themselves, to become more active and more healthy, then that could be of benefit, not necessarily to me, but to the patient." OS9

75% of the clinicians supported Flexifoot measuring compliance to clinical advice.

"It would be really useful in terms of keeping a diary of what your patients are doing, especially with the osteoarthritis patients." PT5

For peer review only - http://bmjopen.bmj.com/site/about/guidelines.xhtml

"It is helpful if you have any doubts as to whether the patients are being compliant, if they are doing too much or too little activity." OS10

Clinicians can use the feedback as a screening tool, and to help determine the next steps for patient management.

"You could use that as some kind of screening test…do they really need to have a knee replacement yet?" OS1

"In conjunction with physiotherapists…so if you were trying to get them to do a particular rehabilitation programme. Monitor what they're doing, that might be very useful." OS6.

However, GPs felt that Flexifoot was a tool to be used more by patients, rather than by GPs for planning patient care: *"an intervention that's positive for the patient, as opposed to this being an investigation" GP2,* and that feedback would be better interpreted by clinicians with greater musculoskeletal knowledge.

"I can't see any acute use for it that's going to change the patient's management right this second now." GP2

"I'm not sure whether this would change my management for the conditions." GP5

Moreover, various participants in all clinician groups agreed that Flexifoot may be more effective not as a stand-alone device, but to enhance current methods for diagnosis and prognosis, as an *"adjunctive thing to what I already have" P3*.

Specific Measurements

Due to the vast array of parameters that can be analysed after Flexifoot use, it was important to determine the most clinically appropriate specific measurements. Participants were prompted by suggestions of symmetry, stride length, centre of pressure, pressure profiles or ground reaction force. The ideal specific measurements differed slightly between clinician groups due to varying levels of knowledge, but there was a convergence of agreed parameters to be measured by Flexifoot in all clinician groups (Figure 2). GPs provided fewer preferable specific measurements due to gaps in their specialist knowledge regarding musculoskeletal disorders.

The clinicians who supported the monitoring of activity levels (type and number of steps) demonstrated its importance for non-OA conditions too.

"It would give you an idea of their daily routine and exercise levels and things, particularly if they've got other conditions such as diabetes or cardiovascular disease which can be improved by exercise." GP3

Two clinicians exhibited a cautious view regarding tracking patients' activity outside of clinical environments:

"You've got to be careful with monitoring… you don't want engender this sort of 'we're watching you' big brother idea." PT3

"I do think that there could be some, a little degree of patients being suspicious of you checking up on them and they may question the clinician – why can't you trust what you're telling them, why do they need to see what I'm doing?" GP2.

Duration of Wear

The duration of Flexifoot wear suggested by clinicians varied from single usage at clinic appointments, up to long-term periods of over one year, implying a range of uses. The nature of the tool being able to offer real-time advice allows for its acute use in the clinic. The prolonged use of Flexifoot may be appropriate for patients with chronic diseases, or post-operatively in those undergoing surgical interventions. The disparity between preferred lengths depended on the rationale for patient use. 7 clinicians revealed that

For peer review only

the duration of Flexifoot wear would be dependent on what outcomes were to be achieved (PT1, PT6, PT8, P2, OS1, OS6), type of injury (PT10), and patient age and compliance (GP5).

"If we were using it as a 'how do they move', we want a picture of their footprint, 5 minutes as they walk along the corridor." PT1

"During periods of activity, if they're experiencing pain or there is a particular challenging part of their day…short bursts of time which are critical to look at, so definitely not all day." OS3

However, long-term monitoring was preferred by most clinicians, where data collection would span over days, weeks, months and years, or *"for as long as it took to establish a meaningful change" PT3.*

"For 24hours or a few days…you'd want the results of this to reflect what they normally do." GP3

A short period of time using the device but at longer intervals between uses was also suggested:

"Snapshots at certain periods, much like we do at follow-up, at 6 weeks, 3 months, and a year." OS4

To maximise the personalised use of Flexifoot, clinicians and patients must collaborate to choose an appropriate duration of wear for each particular case.

Data Presentation

Data Access

50% of the clinicians would prefer to access the data by logging into a system, and some expressed that it should be integrated amongst patients' current notes for information crossover.

"If there was a way of tying in the results so that when you log in and click on the patient, it came up on their results. That would be the most ideal way." GP3

5 clinicians preferred the data via email, although 3 clinicians stated that maintaining patient confidentiality was paramount.

"Emailed is easier, but then log-in would be better because safety aspects and confidentiality." P2

Visual Presentation

The data should be concise so that clinicians can quickly interpret data. The clinicians expressed differing opinions after they were prompted with suggestions of graphs, tables and pictures. Therefore, numerous display options should be available to accommodate for all preferences.

"We need to have a summary that is brief, because you don't want to look through tons and tons of data. Then, if you wanted to look into more detail, then there should be the option." GP4

The data should be easily comprehended by patients too. A visually appealing approach with an additional colour-system scheme can enhance patient understanding.

"Patients want to know as well, they want a variation that's patient friendly…an easier format for patients to understand." OS6.

The addition of normal reference ranges alongside objective measures allows for the comparison of patient parameters versus reference data; this will enhance user-experiences and allow for goals to be set.

"Patient specific – it would be graphs. It would be nice to have a normal distribution and see where they fit inside the normal distribution." OS9

Frequency

The clinicians want to receive and access data at appropriate times, such as immediately before or during appointments. The majority (28/30) of clinicians were unlikely to monitor patients outside of the clinic due to time-restraints.

"I'd access the data just prior to the patient coming in or during that appointment. We would only really have time to monitor or review the data when the patient is actually here." PT5

For peer review only

"Real-time is helpful for the patient in a therapy session. If they had information once a week on how they're doing. Alerts are ideal, but there is no time to check it." PT10

The generation of data should occur in a timely fashion, *"correlated with the patient's clinic appointment" OS9.* The frequency of data received should depend on personal clinician preferences and the purpose of Flexifoot use.

"There should be an option for the data to be accessible when you want it and you choose." GP5

Barriers to Use

Clinicians highlighted obstacles to implementing Flexifoot (Table 2).

Table 2: The barriers to the use of implementing Flexifoot in clinical practice identified by 30 clinicians

**Barriers to the Use of Flexifoot**
• Time
• Cost and availability
• Influence on practice
• Training/education required
• Patient compliance
• Hygiene control

50% of the clinicians illustrated that time restraints were the largest concern of Flexifoot use clinically, including time taken for initial patient assessments, device introduction, and data generation.

"For me to use this for one patient, explain how to use it and monitor their activity is probably unlikely and unrealistic given the general practice workload and increasing demands on GPs." GP2

"That is the difficulty with this, it is an additional investigation that we need to spend time assessing." OS11

The second most identified barrier was cost – *"it has to be suitably priced…that an average practice can afford" P1.*

The inability of clinicians and patients to interpret data and the training required was another issue. 5 GPs stressed that Flexifoot was too specialised for their environment.

"Patients might not understand the data…people are not familiar with technologies, but this will be less of a problem in the future." PT9

"If it's mainly biometrics and gait analysis…I don't think that I would see this as being within a GP's remit so much. The biggest barrier for me not using it is, identifying how the information it gives would fall into my remit, and how it would influence my practice". GP3

Poor patient compliance also hinders Flexifoot's prospective use.

"Patients are so unreliable and I wouldn't be confident that they would remember to transfer it to another pair of shoes or if they take their shoes off and we'd not be tracking anything." OS3

6 clinicians identified hygiene and infection risks if the device was used for long periods, or between different patients. Flexifoot use between multiple patients could be more economically practical however.

For peer review only

"I suppose you've got questions of hygiene…you'd need a material you can wipe and maybe some way of cleaning them really well, so they would be to an infection control standard. If you can use them more than once, it would be cost effective." P3

Future Development

Following the responses to the set questions, clinicians offered suggestions on how to optimise Flexifoot for successful clinical uptake.

Parameters that would be ideally measured using Flexifoot (Figure 2) could be adapted to measure more factors and expand patient target audiences. Clinicians suggested Flexifoot use in Parkinson's disease, peripheral neuropathy in diabetes, chronic pain conditions, obesity, hemiplegia and tendinopathies.

"Pressure profiles are good, but it'd be really great to measure shear and temperatures in the foot…it would be better and more useful for people with plantar foot pain. Diabetics – it would be great." OS11

Design changes were also proposed, *"maybe this ribbon (ankle strap) could be a bit smaller because it might irritate someone on the side and it might artificially affect their gait" OS10.*

DISCUSSION

A technology must be user-friendly to optimise its efficacy and sustainability[16,17]. We conducted structured interviews with clinicians to guide the development of our novel technology, Flexifoot, towards clinical uptake. The clinicians expressed numerous advantages of adopting Flexifoot into healthcare settings and barriers, indicating strategies for future improvement. The main advantage recognised by clinicians was the ability of Flexifoot to generate quantitative data, that can be used for monitoring and feedback in various clinical contexts.

Clinicians implied that Flexifoot would not replace current clinical tools, but instead complement them. The ambulatory quantitative data can support existing OA diagnostic and management tools, and help to improve the reliability of clinical decisions. The ambulatory monitoring of disease progression, alongside patients' responses to treatment, has been considered useful[18]. Other rehabilitation technologies that motivate and offer objective feedback have been associated with long-term benefits and good physical fitness levels[19]. Despite this, some lower limb wearable technologies for rehabilitation have been described to have limited efficacy for the improvement of activity levels, but mainly due to poor research methodologies used in the past[20]. However, there is still a demand for self-management of rehabilitation with feedback using shoe insole pressure sensors[21]. The contradictory results in the literature regarding wearable technologies calls for a study that explores the users' perspectives to maximise acceptance.

Moreover, objective feedback can enable a more efficient exchange and handover of patient information between clinicians[22]. A clinician may use the tool as a screening approach in adjunction to current methods, for more reliable results and subsequently refer the patient onto a specialist[14]. Also, the clinicians recognised that the data can reinforce their dialogue with patients, making patients more aware of their problem. Awareness and feedback was seen as a way to enhance patient self-management. Tracking activity levels and feedback engages patient involvement in their own care, and is useful for other non-musculoskeletal chronic conditions too[23,24].

Clinicians, in our study, indicated how Flexifoot could be a feedback tool for patients. The data being available to patients allows for greater independence in self-monitoring and feedback of their own diseases in familiar environments outside of clinics[18]. Home-based training and monitoring devices showed higher patient satisfaction compared to similar care within clinics[25]. Self-management can also reduce economic burdens as the technology can educate OA patients, improve outcomes and reduce hospital visits[18,26].

The uses and specific measurements suggested by clinicians was greatly dependent on the type of

For peer review only

clinician and specific patient cohorts, which is also the case for other musculoskeletal interventions[27]. The GPs felt that they had insufficient gait analysis knowledge, and that the device was presently too specialist for general practice. GP environments may be inappropriate since it comprises of too broad a range of patients. Instead, they indicated that Flexifoot would be better suited for clinicians that follow up patients more regularly. This was reiterated by physiotherapists, orthopaedic surgeons and podiatrists, who are better equipped in musculoskeletal fields, and expressed positivity for Flexifoot's clinical uptake.

All clinicians indicated that the system and data output should be easy to use and interpret. Ease of use of wearable sensors for clinicians was reported in the past to facilitate their adoption into clinical settings[28]. The prospect of a log-in software system for Flexifoot data was more popular than receiving results via email. The interface should be integrated alongside current patient records for information crossover and a choice of data presentation styles should be available. The material should be presented alongside normal reference ranges, for comparisons and targets to be made. Data accessibility and presentation should be understood by patients too – this is key for patient acceptance and accessibility[29]. Clinicians' views obtained in another study recognised that shorter, simpler and more concise data as educational material is preferable for patients, but that detailed data should be fully available too[30]. This agrees with our findings: participants expressed the possibility of having access to more detailed data if needed, whilst avoiding scanning excessive data beyond their understanding. Full data measurements could be stored however for more skilled users in research settings[31].

Furthermore, shortcomings of Flexifoot were recognised by the clinicians which may explain why the practical application of similar insole monitoring devices have not been successful in the past[32].

The clinicians' continuous workloads means that using real-time data from Flexifoot is only feasible prior to or during appointments in the presence of patients. The real-time data and automatic alerts may be more useful for patient users for receiving feedback, which continues to motivate them[13]**.** Clinicians are reluctant for the introduction of new tools because they can disrupt time-pressured practice schedules, and the time required to train them to use Flexifoot must also be considered[33]**.** In the past, numerous medical wearable technologies for a range of users have failed to meet the criteria of being simple and powerful in terms of data output and energy consumption[32]. However, although clinicians may perceive new tools as a hindrance, a study showed that adults suffering from osteoarthritis felt that more novel approaches could be implemented for the management of their condition[34,35]. The clinical efficacy of Flexifoot must be therefore fully established, such as through patient usability testing, before clinicians can adopt it as a method worthy of appointment time.

High costs also limit new technology implementation within health services. The current expense to manufacture one Flexifoot device is low, but one tool per patient may be economically impractical. The recycling and reuse of devices between patients may reduce costs, but increases hygiene and infection risks, expressed by 6 clinicians. Introducing hygienic procedures prior to and after Flexifoot use could enhance its reusability and cost-effectiveness. Thus, the cost-effectiveness, with reference to current treatment guidelines, should be further investigated. Patient compliance is also an issue since long-term wearable devices require adequate patient acceptability. However, from a similar study we conducted that explored patients' views, all participants were keen for the uptake of wearable technologies[13]. Moreover, effective clinician-patient communication can determine patient cohorts supportive of self-management, and those more likely to adhere to using Flexifoot[13,14,36]. Clinicians can promote the relevance of sensor technologies for patients' care, and hence boost compliance[12,37]. The identified issues surrounding Flexifoot are apparent in other wearable technologies too[27]. The replication of problems between devices implies a necessity for new approaches in encouraging patients' compliance and appeal for novel strategies. The findings that emerged from this study can be translated to other similar technologies to promote their clinical uptake and foster new developments.

The study limitations involved the clinicians' varied levels of experience and familiarity of instrumented insoles, and that 27/30 interviewees were based in the London area. The clinicians had not used Flexifoot, and telephone interviews could not view the device, but detailed descriptions and

For peer review only

commercially similar devices that could be found online were provided prior to interviews. Future studies would involve clinicians' use of Flexifoot beforehand. Moreover, clinicians who were well-informed around the subject were perhaps biased to initially participate and express positivity. However, the interviews were confidential and honest feedback was encouraged.

In conclusion, the clinicians considered Flexifoot to be a useful tool that could be used in adjunction to current approaches, in a long-term, follow-up setting to support and improve patient care. The clinicians' preferences exhibited numerous ways in which Flexifoot can be useful for patients with OA or other conditions. The measured parameters should be selected according to patient-specific cases, and delivered in a concise manner through a secure interface. A choice of data outputs should be offered to cater for all users. The challenges of time, cost, infection control should be addressed, alongside the clinical efficacy and cost-effectiveness for the clinical adoption of Flexifoot and similar technologies.

Author Contributions: DL, EP, and AHM conceived and designed the study. DL carried out the interviews; DL and EP analysed and interpreted the data. DL and EP drafted the manuscript. All authors read, edited, and approved the final version of the manuscript. All authors had full access to all of the data in the study and can take responsibility for the integrity of the data and the accuracy of the data analysis.

Acknowledgement: We would like to thank MSc student Mary Goodwin for her contribution with data collection.

Funding: This research received no specific grant from any funding agency in the public, commercial or not-for-profit sectors

Competing interests: The authors declare that there are no conflicts of interest.

All authors have completed the ICMJE uniform disclosure form at www.icmje.org/coi_disclosure.pdf (available on request from the corresponding author) and declare: no support from any organisation for the submitted work; no financial relationships with any organisations that might have an interest in the submitted work in the previous three years; no other relationships or activities that could appear to have influenced the submitted work.

Ethical approval: The study was reviewed and approved by Imperial College London Ethics Research Committee. All participants gave written informed consent.

Data sharing: No additional data available.

References

1 Bennell KL, Hunter DJ, Hinman RS. Management of osteoarthritis of the knee. *Bmj* 2012;**345**:e4934–e4934. doi:10.1136/bmj.e4934

2 Simon D, Mascarenhas R, Saltzman BM, *et al.* The Relationship between Anterior Cruciate Ligament Injury and Osteoarthritis of the Knee. *Adv Orthop* 2015;**2015**. doi:10.1155/2015/928301

3 Muthuri SG, McWilliams DF, Doherty M, *et al.* History of knee injuries and knee osteoarthritis: A meta-analysis of observational studies. *Osteoarthr Cartil* 2011;**19**:1286–93. doi:10.1016/j.joca.2011.07.015

4 Driban JB, Eaton CB, Lo GH, *et al.* Association of Knee Injuries With Accelerated Knee Osteoarthritis Progression: Data From the Osteoarthritis Initiative. *Arthritis Care Res* 2014;**66**:1673–9. doi:10.1002/acr.22359

5 Jones CA, Voaklander DC, Johnston DW, *et al.* Health related quality of life outcomes after total hip and knee arthroplasties in a community based population. *J Rheumatol* 2000;**27**:1745–52.http://www.ncbi.nlm.nih.gov/pubmed/10914862 (accessed 29 Mar 2018).

6 Wylde V, Hewlett S, Learmonth ID, *et al.* Persistent pain after joint replacement: Prevalence, sensory qualities, and postoperative determinants. *Pain* 2011;**152**:566–72. doi:10.1016/j.pain.2010.11.023

7 Singh JA, O'Byrne M, Harmsen S, *et al.* Predictors of moderate-severe functional limitation after primary Total Knee Arthroplasty (TKA): 4701 TKAs at 2-years and 2935 TKAs at 5-years. *Osteoarthr Cartil* 2010;**18**:515–21. doi:10.1016/j.joca.2009.12.001

For peer review only

8 Dowsey MM, Nikpour M, Dieppe P, *et al.* Associations between pre-operative radiographic changes and outcomes after total knee joint replacement for osteoarthritis. *Osteoarthr Cartil* 2012;**20**:1095–102. doi:10.1016/j.joca.2012.05.015

9 Elbaz A, Mor A, Segal G, *et al.* Novel classification of knee osteoarthritis severity based on spatiotemporal gait analysis. *Osteoarthr Cartil* 2014;**22**:457–63. doi:10.1016/j.joca.2013.12.015

10 Harris KK, Dawson J, Jones LD, *et al.* Extending the use of PROMs in the NHS—using the Oxford Knee Score in patients undergoing non-operative management for knee osteoarthritis: a validation study. *BMJ Open* 2013;**3**:e003365. doi:10.1136/bmjopen-2013-003365

11 Roos EM, Roos HP, Lohmander LS, *et al.* Knee Injury and Osteoarthritis Outcome Score (KOOS)—Development of a Self-Administered Outcome Measure. *J Orthop Sport Phys Ther* 1998;**28**:88–96. doi:10.2519/jospt.1998.28.2.88

12 Bergmann JHM, McGregor AH. Body-worn sensor design: What do patients and clinicians want? *Ann Biomed Eng* 2011;**39**:2299–312. doi:10.1007/s10439-011-0339-9

13 Papi E, Belsi A, McGregor AH. A knee monitoring device and the preferences of patients living with osteoarthritis: a qualitative study. *BMJ Open* 2015;**5**:e007980. doi:10.1136/bmjopen-2015-007980

14 Papi E, Murtagh GM, McGregor AH. Wearable technologies in osteoarthritis: a qualitative study of clinicians' preferences. *BMJ Open* 2016;**6**:e009544. doi:10.1136/bmjopen-2015-009544

15 Ritchie J, Lewis J. QUALITATIVE RESEARCH PRACTICE A Guide for Social Science Students and Researchers. https://mthoyibi.files.wordpress.com/2011/10/qualitative-research-practice_a-guide-for-social-science-students-and-researchers_jane-ritchie-and-jane-lewis-eds_20031.pdf (accessed 13 Dec 2017).

16 Lewis JE, Neider MB. Designing Wearable Technology for an Aging Population. *Ergon Des* 2017;**25**:4–10. doi:10.1177/1064804616645488

17 Lee J, Kim D, Ryoo H-Y, *et al.* Sustainable Wearables: Wearable Technology for Enhancing the Quality of Human Life. *Sustainability* 2016;**8**:466. doi:10.3390/su8050466

18 Majumder S, Mondal T, Deen M. Wearable Sensors for Remote Health Monitoring. *Sensors* 2017;**17**:130. doi:10.3390/s17010130

19 Aamot I-L, Karlsen T, Dalen H, *et al.* Long-term Exercise Adherence After High-intensity Interval Training in Cardiac Rehabilitation: A Randomized Study. *Physiother Res Int* 2016;**21**:54–64. doi:10.1002/pri.1619

20 Powell L, Parker J, Martyn St-James M, *et al.* The Effectiveness of Lower-Limb Wearable Technology for Improving Activity and Participation in Adult Stroke Survivors: A Systematic Review. *J Med Internet Res* 2016;**18**:e259. doi:10.2196/jmir.5891

21 Munoz-Organero M, Parker J, Powell L, *et al.* Assessing Walking Strategies Using Insole Pressure Sensors for Stroke Survivors. *Sensors (Basel)* 2016;**16**. doi:10.3390/s16101631

22 Popovici I, Morita PP, Doran D, *et al.* Technological aspects of hospital communication challenges: An observational study. *Int J Qual Heal Care* 2015;**27**:183–8. doi:10.1093/intqhc/mzv016

23 Chiauzzi E, Rodarte C, DasMahapatra P. Patient-centered activity monitoring in the self-management of chronic health conditions. *BMC Med* 2015;**13**:1–6. doi:10.1186/s12916-015-0319-2

24 Thornton JS, Frémont P, Khan K, *et al.* Physical activity prescription: a critical opportunity to address a modifiable risk factor for the prevention and management of chronic disease: a position statement by the Canadian Academy of Sport and Exercise Medicine. *Br J Sports Med* 2016;**50**:1109–14. doi:10.1136/bjsports-2016-096291

25 Kraal JJ, Elske Van Den Akker-Van Marle M, Abu-Hanna A, *et al.* Clinical and cost-effectiveness of home-based cardiac rehabilitation compared to conventional, centre-based cardiac rehabilitation: Results of the FIT@Home study. *Eur J Prev Cardiol* 2017;**24**:1260–73. doi:10.1177/2047487317710803

26 Bodenheimer T, Lorig K. Patient Self-management of Chronic Disease in Primary Care. 2014;**288**:2469–75. doi:10.1001/jama.288.19.2469

27 de Almeida PHTQ, MacDermid JC, Pontes TB, *et al.* Orthotic use for CMC osteoarthritis: Variations among different health professionals in Brazil. *J Hand Ther* 2016;**29**:440–50. doi:10.1016/j.jht.2016.07.007

28 Grimm B, Bolink S. Evaluating physical function and activity in the elderly patient using wearable motion sensors. *EFORT Open Rev* 2016;**1**:112–20. doi:10.1302/2058-5241.1.160022

For peer review only

29 Puri A, Kim B, Nguyen O, *et al.* User Acceptance of Wrist-Worn Activity Trackers Among Community-Dwelling Older Adults: Mixed Method Study. *JMIR mHealth uHealth* 2017;**5**:e173. doi:10.2196/mhealth.8211

30 Grudniewicz A, Bhattacharyya O, McKibbon KA, *et al.* User-centered design and printed educational materials: A focus group study of primary care physician preferences. *J Contin Educ Health Prof* 2016;**36**:249–55. doi:10.1097/CEH.0000000000000112

31 Iqbal MH, Aydin A, Brunckhorst O, *et al.* A review of wearable technology in medicine. *J R Soc Med* 2016;**109**:372–80. doi:10.1177/0141076816663560

32 Haghi M, Thurow K, Stoll R. Wearable Devices in Medical Internet of Things: Scientific Research and Commercially Available Devices. *Healthc Inform Res* 2017;**23**:4–15. doi:10.4258/hir.2017.23.1.4

33 Yarbrough a. K, Smith TB. A new take on TAM. *Med Care Res Rev* 2007;**64**:650–72. doi:10.1177/1077558707305942

34 Mackay C, Hawker GA, Jaglal SB. Qualitative study exploring the factors influencing physical therapy management of early knee osteoarthritis in Canada. *BMJ Open* 2018;**8**:23457. doi:10.1136/bmjopen-2018-023457

35 MacKay C, Sale J, Badley EM, *et al.* Qualitative Study Exploring the Meaning of Knee Symptoms to Adults Ages 35-65 Years. *Arthritis Care Res (Hoboken)* 2016;**68**:341–7. doi:10.1002/acr.22664

36 Loos JR, Davidson EJ. Wearable health monitors and physician-patient communication: The physician's perspective. *Proc Annu Hawaii Int Conf Syst Sci* 2016;**2016–March**:3389–98. doi:10.1109/HICSS.2016.422

37 Teh RC-A, Mahajan N, Visvanathan R, *et al.* Clinical effectiveness of and attitudes and beliefs of health professionals towards the use of health technology in falls prevention among older adults. *Int J Evid Based Healthc* 2015;**13**:213–23. doi:10.1097/XEB.0000000000000029

Supplementary files: Semi-structured interview questions for clinicians

Figure legends:

Figure 1: a) Layout of pressure sensors on the insoles with connectors for the circuit boards. b) Insoles covered with neoprene with circuit boards for data transmission attached and ready to be inserted into shoes.

Figure 2: Clinician preferences for the specific measurements that they would like Flexifoot to obtain.

For peer review only

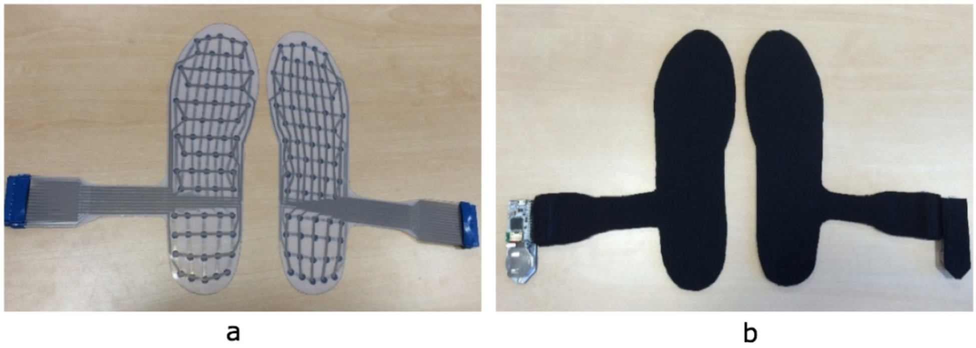

Figure 1: a) Layout of pressure sensors on the insoles with connectors for the circuit boards. b) Insoles covered with neoprene with circuit boards for data transmission attached and ready to be inserted into shoes.

157x62mm (200 x 200 DPI)

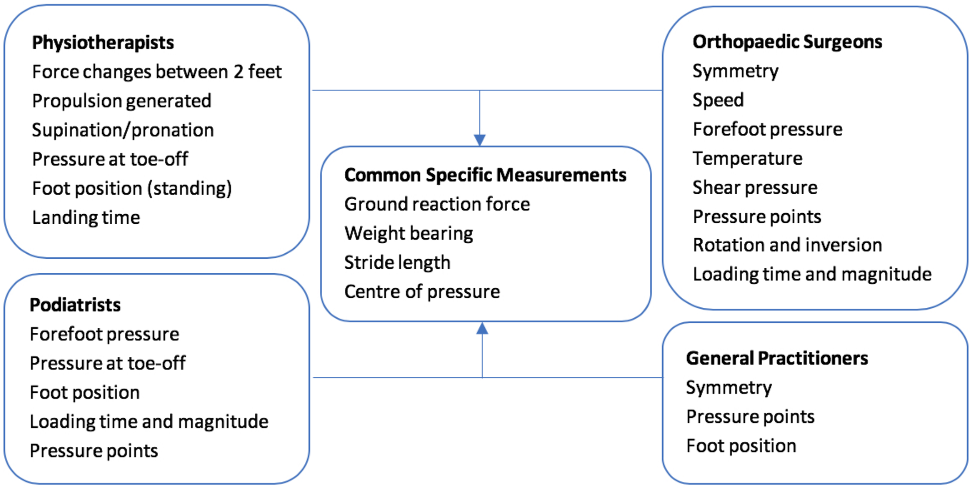

Figure 2: Clinician preferences for the specific measurements that they would like Flexifoot to obtain.

152x74mm (200 x 200 DPI)

Semi-structured interview questions for clinicians

Contextual Background

- What is your age and nationality (you can prefer not to say)?
- What is/can you describe your professional role?
- How long have you been in this role?
- What types of patients do you come into contact with?
    A. Is there a particular type/category e.g OA/ACL?
    B. Does it vary?
    C. How many patients do you see per day?
    D. Is there a particular age range of patients you work with?

Wearable Technologies

- What do you know about wearable technologies?
- Do you currently use any wearable technologies in your work?

Clinician Preferences for the Flexifoot Device (With Reference to OA Surgery and ACL Injuries)

- Would you find Flexifoot useful for your OA and/or ACL injury patients?
- How would such a device help you in your own work? How would you use it?
- What would you specifically like to measure using the Flexifoot device?
- Would you like information about e.g.
    A. the patient's gait, such as symmetry/stride length?
    B. centre of pressure and pressure profiles for subsections of the foot?
    C. ground reaction force?
- Is there any parameters that should be tailored for OA surgery or for ACL injuries patients? (Different measurements used in accordance to the patients?)
- In addition to specific parameters, would you find it useful to measure the activity level of your patients? (e.g. time when active or not)
- How do you feel about monitoring compliance to exercise programmes?
- How often and for how long would you want the patient to wear the device?
- How would you like the data presented to you? E.g. in graphs, summary tables, performance profiles over a certain period of time?
- Would you find it useful to have a brief summary of the patient's progress with the option to look in more detail at certain aspects of the data?
- When would you like the data to be available to you? E.g. every day the patient uses Flexifoot?
- How would you ideally access the data? Would you like it emailed to you or would you prefer to have a website where you can log in to access it?
- Would you find an automatic alert system useful that told you when the data was available from your patient?
- Would you like an alert that will flag up if the metrics you identified fall below a certain threshold? (e.g. the patient is not doing exercise at all, so you need to send them a reminder?)
- Would you find Flexifoot to be more useful for patients with ACL injury or for pre-/post-OA surgery?
- What other information would you use to complement the use of Flexifoot in your clinical practice? Any other parameters that you think will be valuable that cannot be measured with Flexifoot?
- Can you suggest any reasons that may prevent you from using this device should it became available for use in clinical practice?

Closure

- Do you have any other comments about the Flexifoot device?

For peer review only

Standards for Reporting Qualitative Research (SRQR)*

http://www.equator-network.org/reporting-guidelines/srqr/

Title and abstract

	Page/line no(s).
**Title** - Concise description of the nature and topic of the study Identifying the study as qualitative or indicating the approach (e.g., ethnography, grounded theory) or data collection methods (e.g., interview, focus group) is recommended	Page 1: Lines 1-2
**Abstract** - Summary of key elements of the study using the abstract format of the intended publication; typically includes background, purpose, methods, results, and conclusions	Page 1: Lines 25-50

Introduction

**Problem formulation** - Description and significance of the problem/phenomenon studied; review of relevant theory and empirical work; problem statement	Page 2: Lines 15-51
**Purpose or research question** - Purpose of the study and specific objectives or questions	Page 2: Lines 49-54<br>Page 3: Lines 1-6

Methods

**Qualitative approach and research paradigm** - Qualitative approach (e.g., ethnography, grounded theory, case study, phenomenology, narrative research) and guiding theory if appropriate; identifying the research paradigm (e.g., postpositivist, constructivist/ interpretivist) is also recommended; rationale**	Page 3: Line 10-19,<br>Page 3: Lines 5-24-43
**Researcher characteristics and reflexivity** - Researchers' characteristics that may influence the research, including personal attributes, qualifications/experience, relationship with participants, assumptions, and/or presuppositions; potential or actual interaction between researchers' characteristics and the research questions, approach, methods, results, and/or transferability	Page 3: Lines 25-28<br>Page 10: Lines 5-6
**Context** - Setting/site and salient contextual factors; rationale**	Page 3: Lines 11-12, 16-22, 40-43
**Sampling strategy** - How and why research participants, documents, or events were selected; criteria for deciding when no further sampling was necessary (e.g., sampling saturation); rationale**	Page 2: Lines 53-54<br>Page 3: Lines 1-4, 40-43
**Ethical issues pertaining to human subjects** - Documentation of approval by an appropriate ethics review board and participant consent, or explanation for lack thereof; other confidentiality and data security issues	Page 3: Lines 11-13, 40-43
**Data collection methods** - Types of data collected; details of data collection procedures including (as appropriate) start and stop dates of data collection and analysis, iterative process, triangulation of sources/methods, and modification of procedures in response to evolving study findings; rationale**	Page 3: Line 11, 24-37

For peer review only - http://bmjopen.bmj.com/site/about/guidelines.xhtml

**Data collection instruments and technologies** - Description of instruments (e.g., interview guides, questionnaires) and devices (e.g., audio recorders) used for data collection; if/how the instrument(s) changed over the course of the study	Page 3: Lines 11, 24-37
**Units of study** - Number and relevant characteristics of participants, documents, or events included in the study; level of participation (could be reported in results)	Page 3: Lines 15-33, 46-48
**Data processing** - Methods for processing data prior to and during analysis, including transcription, data entry, data management and security, verification of data integrity, data coding, and anonymization/de-identification of excerpts	Page 3: Line 11, 24-37
**Data analysis** - Process by which inferences, themes, etc., were identified and developed, including the researchers involved in data analysis; usually references a specific paradigm or approach; rationale**	Page 3: Lines 24-37, 46-51<br>Page 4: 1-2
**Techniques to enhance trustworthiness** - Techniques to enhance trustworthiness and credibility of data analysis (e.g., member checking, audit trail, triangulation); rationale**	Page 3: Lines 34-37

Results/findings

**Synthesis and interpretation** - Main findings (e.g., interpretations, inferences, and themes); might include development of a theory or model, or integration with prior research or theory	Page 3: Lines 46-51<br>Page 4: Lines 1-3
**Links to empirical data** - Evidence (e.g., quotes, field notes, text excerpts, photographs) to substantiate analytic findings	Page 4: Lines 6-42<br>Pages 5-7<br>Page 8: Lines 1-10

Discussion

**Integration with prior work, implications, transferability, and contribution(s) to the field -** Short summary of main findings; explanation of how findings and conclusions connect to, support, elaborate on, or challenge conclusions of earlier scholarship; discussion of scope of application/generalizability; identification of unique contribution(s) to scholarship in a discipline or field	Page 8: Lines 13-52<br>Page 9: Lines 1-50
**Limitations** - Trustworthiness and limitations of findings	Page 9: Lines 35-41

Other

**Conflicts of interest** - Potential sources of influence or perceived influence on study conduct and conclusions; how these were managed	Page 10: Lines 7-12
**Funding** - Sources of funding and other support; role of funders in data collection, interpretation, and reporting	Page 10: Lines 5-6

For peer review only - http://bmjopen.bmj.com/site/about/guidelines.xhtml

*The authors created the SRQR by searching the literature to identify guidelines, reporting standards, and critical appraisal criteria for qualitative research; reviewing the reference lists of retrieved sources; and contacting experts to gain feedback. The SRQR aims to improve the transparency of all aspects of qualitative research by providing clear standards for reporting qualitative research.

**The rationale should briefly discuss the justification for choosing that theory, approach, method, or technique rather than other options available, the assumptions and limitations implicit in those choices, and how those choices influence study conclusions and transferability. As appropriate, the rationale for several items might be discussed together.

Reference:

O'Brien BC, Harris IB, Beckman TJ, Reed DA, Cook DA. **Standards for reporting qualitative research: a synthesis of recommendations.** *Academic Medicine*, Vol. 89, No. 9 / Sept 2014 DOI: 10.1097/ACM.0000000000000388

For peer review only

For peer review only - http://bmjopen.bmj.com/site/about/guidelines.xhtml
